# Superior canal dehiscence syndrome induces canal-specific kinematic adaptations during locomotion

**DOI:** 10.1038/s41598-025-16904-7

**Published:** 2025-09-29

**Authors:** Raabeae Aryan, Jennifer L. Millar, Chenhao Bao, John P. Carey, Michael C. Schubert, Kathleen E. Cullen

**Affiliations:** 1 Department of Biomedical Engineering, Johns Hopkins University School of Medicine, Baltimore, MD USA; 2 Department of Physical Medicine and Rehabilitation, Johns Hopkins University School of Medicine, Baltimore, MD USA; 3 Department of Otolaryngology-Head and Neck Surgery, Johns Hopkins University School of Medicine, Baltimore, MD USA; 4 Department of Neuroscience, Johns Hopkins University School of Medicine, Baltimore, MD USA; 5 Kavli Neuroscience Discovery Institute, Johns Hopkins University, Baltimore, MD USA

**Keywords:** Superior canal dehiscence syndrome, Vestibular system, Gait, Rehabilitation, Diseases, Motor control

## Abstract

Superior canal dehiscence syndrome (SCDS) is caused by a pathological ‘third window’ in the inner ear that selectively impairs superior semicircular canal function. Here we examined locomotion in individuals with SCDS to explore whether this canal-specific vestibular disruption leads to distinctive changes in movement during natural, overground walking. Participants with unilateral SCDS and healthy controls completed a series of ten walking tasks of varying difficulty (i.e., Functional Gait Assessment), while wearing inertial sensors on the head, trunk, waist, and limbs to capture segment-specific body movements. Participants with SCDS showed significantly lower FGA scores and slower gait cycles, as well as movement changes primarily in the vertical and pitch planes. In particular, quantitative analysis of kinematics revealed reduced vertical head acceleration with increased variability during complex tasks, diminished head pitch velocity, and reduced ipsi-lesional ankle pitch velocity and vertical acceleration. Importantly, these alterations were most pronounced during challenging tasks with limited visual feedback and could not be explained by slower gait speed alone. Overall, our findings suggest that disruption of a single semicircular canal can elicit compensatory movement strategies that reduce stimulation of the affected canal, thereby offering new insight into canal-specific contributions to everyday mobility and informing targeted vestibular rehabilitation.

## Introduction

Superior canal dehiscence (SCD) is a condition resulting from an abnormal opening in the bony structure surrounding the superior semicircular canal of the vestibular organ^1^. This pathological third window disrupts the normal transmission of pressure and sound within the inner ear^2,3^, leading to a range of auditory and vestibular symptoms collectively known as superior canal dehiscence syndrome (SCDS). Affected individuals often experience sound- and pressure-induced vertigo, and nystagmus in the plane of the impaired semicircular canal in response to loud noise or pressure changes^4,5^. As a result, individuals with SCDS frequently report dizziness, oscillopsia, autophony, and chronic imbalance^4–7^, which can significantly impact their quality of life^8,9^.

Despite the well-documented vestibular and auditory disturbances in SCDS, it is unknown whether and how this condition alters movement in everyday life. Locomotion offers an ideal window into this question, as it requires the coordination of gaze, head and postural control—functions in which the vestibular system plays an essential role. Through reflex pathways such as the vestibulo-ocular, vestibulo-collic, and vestibulo-spinal reflexes, the vestibular system stabilizes gaze and maintains postural orientation during our everyday activities^10–12^. While prior work has shown a weak negative correlation between the length of the dehiscence and pre-operative VOR gain for the affected superior canal^13^, the functional consequences for everyday movements including walking remain poorly understood. The objective assessments of head and postural kinematics during walking would provide key insights into how SCDS shapes movement strategies, yet such data are sparse. Indeed, to date, only one study has evaluated functional gait in SCDS, reporting normal scores on the Dynamic Gait Index (DGI)^14^. However, metrics like the DGI offer limited insight into the specific movement adaptations that may emerge when the kinematics of body segments are recorded during performance-based measures under vestibular challenge.

Accordingly, here to address this gap, we investigated whether SCDS alters head and postural kinematics during gait. Specifically, we hypothesized that head pitch velocity and vertical acceleration—measures corresponding to motion with significant projection in the plane of the superior semicircular canal—would be most affected. Using a wearable-based kinematic approach previously validated in other vestibular disorders^15,16^ we quantified head, trunk, and limb motions in individuals with unilateral SCDS and healthy controls while performing each item (i.e., task) of the Functional Gait Assessment (FGA) scale. Inertial measurement units (IMUs) were placed on the head, back, waist, dominant wrist, and lower extremities to record three-dimensional angular velocity and linear acceleration. Additionally, we explored whether a simplified kinematic approach—using a single IMU and a reduced set of gait tasks—could effectively distinguish individuals with SCDS from controls. Finally, we examined correlations between kinematic measures and clinical outcomes to determine whether specific gait alterations in affected individuals relate to broader functional, and perceived impairments.

Overall, our findings demonstrated that individuals with SCDS adopt distinct gait adaptations, including significantly reduced vertical head accelerations, lower head pitch velocities, and slower gait speeds across tasks with high sensory and motor demands. These changes likely reflect compensatory strategies to minimize stimulation of the affected superior canal and reduce symptom severity. By establishing the broader impact of SCDS on movement during locomotion, these results provide insights that may guide rehabilitation strategies aimed at improving stability and mobility in affected individuals.

## Methods

This prospective study was approved by the Institutional Review Board of the Johns Hopkins Hospital (IRB00246479) and conducted in accordance with the institutional guidelines for safe and ethical research in human subjects.

### Participants

An a-priori power analysis was not performed for sample size estimation in this study, and a convenience sample of eligible individuals who had attended the Department of Otolaryngology-Head and Neck Surgery tertiary medical center at the Johns Hopkins Hospital to receive SCDS-related medical care, between February 2022 to November 2023, were recruited. Sixteen individuals (mean age = 46.8 ± 11.2 years) with unrepaired, unilateral SCD syndrome (SCDS), who were diagnosed as having unilateral symptoms according to the Bárány Society diagnostic criteria^4^ were included (including 8 with unilateral and 8 with bilateral evidence of bony dehiscence observed on the computed tomography imaging; demographic and clinical characteristics of both sub-groups are presented in Table 2). Individuals with SCDS were excluded if they were representing clinical evidence of bilateral Bárány SCD syndrome or had previously received any type of surgical interventions on either ear. Individuals with bilateral symptoms (bilateral Bárány SCDS) were excluded to avoid overestimating group effects, as bilateral Bárány SCDS cases often present with more pronounced symptoms and vestibular impairment than unilateral Bárány SCDS. Additionally, 16 age- and sex-matched healthy individuals who had no history of neurologic or otologic conditions participated in this study (mean age = 47.3 ± 11.0 years) (Table 1). In general, individuals in both groups were deemed ineligible if they had any acute and/or medically unmanaged migraine, were unable to ambulate independently, or had any other pathologies impacting their mobility and postural stability. Written informed consents were obtained from both the SCDS and control groups prior to the data collection. Additionally, participants were instructed to wear their comfortable walking shoes on the assessment day.

**Table 1:** Participants demographic and clinical characteristics.

Clinical measures	SCDS group	Control group	*p* value
Age (years)	46.8 ± 11.2	47.3 ± 11.0	*ns*
Sex (M, F)	6, 10	6, 10	
Height (cm)	169.8 ± 9.7	168.4 ± 10.1	*ns*
Weight (lbs.)	188.6 ± 40.9	168.9 ± 39.2	*ns*
Symptomatic side (R, L)	6, 10	*NA*	–
FGA (score)	25.19 ± 3.66	28.81 ± 1.17	***
TUG (s)			
Ipsi-lesional	10.24 ± 1.27	7.96 ± 1.34	***
Contra-lesional	10.05 ± 1.37	8.04 ± 1.20	***
TUG.Cog (s)			
Ipsi-lesional	12.82 ± 3.68	9.67 ± 1.77	**
Contra-lesional	13.14 ± 3.73	9.50 ± 1.73	**
Gait speed (m/s)	1.22 ± 0.17	1.41 ± 0.16	**
ABC (%)	87.09 ± 13.06	97.20 ± 4.70	**
OFI (score)	48.75 ± 24.76	15.47 ± 7.00	***
PPPD (score)	16.27 ± 11.90	2.00 ± 2.56	***
DHI (score)	31.50 ± 19.86	2.00 ± 4.56	***
HIT-6 (score)	48.88 ± 8.86	43.50 ± 8.63	*ns*
BAI (score)	13.88 ± 9.91	4.38 ± 7.92	**
Autophony^†^ (score)	32.13 ± 27.55	0	***
LogMAR (score)			
Static	− 0.14 ± 0.09	− 0.11 ± 0.11	*ns*
Ipsi-Horizontal^‡^	0.27 ± 0.13	0.21 ± 0.09	*ns*
Contra-Horizontal^‡^	0.31 ± 0.15	0.21 ± 0.10	*
Upward^‡^	0.26 ± 0.14	0.21 ± 0.12	*ns*
Downward^‡^	0.31 ± 0.13	0.19 ± 0.12	*
vHIT VOR Gain			
Ipsi-Horizontal	0.95 ± 0.05	0.98 ± 0.10	*ns*
Ipsi-Posterior	0.73 ± 0.20	0.76 ± 0.11	*ns*
Ipsi-Anterior	0.66 ± 0.18	0.94 ± 0.16	***
Contra-Horizontal	0.97 ± 0.07	0.92 ± 0.08	*ns*
Contra-Posterior	0.76 ± 0.17	0.90 ± 0.13	*
Contra-Anterior	0.78 ± 0.25	0.72 ± 0.17	*ns*

FGA, Functional Gait Assessment; TUG, Timed Up and Go; TUG.Cog, Timed Up and Go while counting backwards by three; ABC, Activities-specific Balance Confidence questionnaire; OFI, Oscillopsia Functional Impact; PPPD, Niigata Persistent Postural-Perceptual dizziness questionnaire; DHI, Dizziness Handicap Inventory; HIT-6, Headache Impact Test.; BAI, Beck Anxiety Inventory; Ipsi, Denotes the symptomatic side for SCDS group, and right side for control group; Contra, Denotes the asymptomatic side for SCDS group, and left side for control group; LogMAR, (Logarithm of the minimum angle of resolution) Logarithmic visual acuity score based on the dynamic visual acuity (DVA) test; vHIT, video Head Impulse Test; VOR, Vestibulo-ocular reflex; NA, not assessed/not applicable. Presented values are means ± standard deviations for continuous variables, and counts for categorical variables. Significance level α = 0.05. ns: not significant. *p < 0.05, **p < 0.01, ***p < 0.001. ^**†**^Autophony was measured in 8 SCDS and 7 healthy individuals. ^**‡**^Corrected LogMAR score values.

**Table 2:** Demographic and clinical characteristics of unilateral anatomical SCD and bilateral anatomical SCD sub-groups.

Clinical measures	Unilateral anatomical SCD group (n = 8)	Bilateral anatomical SCD group (n = 8)	*p* value
Age (years)	50.62 ± 13.82	42.88 ± 6.58	*ns*
Sex (M, F)	3,5	3,5	–
Height (cm)	169.49 ± 9.42	170.20 ± 10.54	*ns*
Weight (lbs.)	184.50 ± 38.73	192.62 ± 45.19	*ns*
Symptomatic side (R, L)	4,4	2,6	–
FGA (score)	25.50 ± 3.42	24.88 ± 4.09	*ns*
TUG (s)			
Ipsi-lesional	10.06 ± 0.99	10.42 ± 1.55	*ns*
Contra-lesional	9.85 ± 1.24	10.25 ± 1.55	*ns*
TUG.Cog (s)			
Ipsi-lesional	13.06 ± 4.76	12.57 ± 2.55	*ns*
Contra-lesional	13.05 ± 4.79	13.22 ± 2.67	*ns*
Gait speed (m/s)	1.19 ± 0.21	1.24 ± 0.13	*ns*
ABC (%)	86.05 ± 14.62	88.13 ± 12.23	*ns*
OFI (score)	43.25 ± 19.03	54.25 ± 29.70	*ns*
PPPD (score)	16.14 ± 11.80	16.38 ± 12.81	*ns*
DHI (score)	28.25 ± 22.21	34.75 ± 18.11	*ns*
HIT-6 (score)	50.25 ± 8.68	47.50 ± 9.41	*ns*
BAI (score)	15.25 ± 12.87	12.50 ± 6.35	*ns*
Autophony^†^ (score)	47.75 ± 30.37	16.50 ± 14.06	*NA*
LogMAR (score)			
Static	− 0.17 ± 0.09	− 0.11 ± 0.08	*ns*
Ipsi-Horizontal^‡^	0.28 ± 0.15	0.26 ± 0.12	*ns*
Contra-Horizontal^‡^	0.38 ± 0.19	0.25 ± 0.07	*ns*
Upward^‡^	0.28 ± 0.14	0.24 ± 0.15	*ns*
Downward^‡^	0.39 ± 0.14	0.25 ± 0.05	*ns*
vHIT VOR Gain			
Ipsi-Horizontal	0.95 ± 0.05	0.95 ± 0.06	*ns*
Ipsi-Posterior	0.79 ± 0.14	0.69 ± 0.25	*ns*
Ipsi-Anterior	0.62 ± 0.13	0.69 ± 0.21	*ns*
Contra-Horizontal	0.97 ± 0.08	0.97 ± 0.06	*ns*
Contra-Posterior	0.75 ± 0.13	0.77 ± 0.20	*ns*
Contra-Anterior	0.83 ± 0.20	0.74 ± 0.30	*ns*

SCD, Superior Canal Dehiscence; FGA, Functional Gait Assessment; TUG, Timed Up and Go; TUG.Cog, Timed Up and Go while counting backwards by three; ABC, Activities-specific Balance Confidence questionnaire; OFI, Oscillopsia Functional Impact; PPPD, Niigata Persistent Postural-Perceptual dizziness questionnaire; DHI, Dizziness Handicap Inventory; HIT-6, Headache Impact Test; BAI, Beck Anxiety Inventory; Ipsi, Denotes the symptomatic side; Contra, Denotes the asymptomatic side; LogMAR, (Logarithm of the minimum angle of resolution) Logarithmic visual acuity score based on the dynamic visual acuity (DVA) test; vHIT, video Head Impulse Test; VOR, Vestibulo-ocular reflex; NA, not assessed/not applicable. Presented values are means ± standard deviations for continuous variables, and counts for categorical variables. Significance level α = 0.05. ns: not significant. *p < 0.05, **p < 0.01, ***p < 0.001. ^**†**^Autophony index data was available for 4/8 unilateral anatomical SCD and 4/8 bilateral anatomical SCD participants; therefore, due to the low power and large within-groups variability, a statistical comparison of the autophony index was not performed between sub-groups. ^**‡**^Corrected LogMAR score values.

### Clinical measures

The following clinical outcome measures were evaluated (Table 1):

- *Physiological measures* To examine the effectiveness of the semicircular canals in response to passive head rotations, we performed the video head impulse test^17^ to measure the gains of the VOR reflex along all 3 semicircular canal planes of both ears in both participant groups (vHIT; ICS, Otometrics/Natus Medical Incorporated, Denmark).
- *Functional measures* In both groups, the Dynamic Visual Acuity test (DVA)^18^ was used to assess the efficacy of the VOR reflex during active horizontal and vertical sinusoidal head rotations, with visual fixation on a target displayed on a tablet screen 2 m away. Results of the DVA test were reported as ‘corrected’ LogMAR scores (logarithm of the minimum angle of resolution)^19^, calculated by subtracting the dynamic visual acuity scores from the static visual acuity score. Ipsi- and contra-lesional refer to yaw head rotations. Gait performance in both groups was quantified by using: (a) individuals’ normal gait speed, calculated over a 10-m walkway; (b) Timed Up and Go (TUG) test^20^, once performed as a single-task TUG (with turning around the cone towards both ipsi- and contra-lesional sides), and also as a cognitive dual-task (while counting backwards by three); and (c) the Functional Gait Assessment scale (FGA)^21,22^. The specific tasks of the FGA scale are presented in Table 3.
- *Participant-reported outcome measures (PROMs)* In order to determine participants’ self-perceived balance confidence, anxiety level, headache, severity of postural dizziness, and dizziness-induced disability during daily life activities, both groups of participants completed the Activities-specific Balance Confidence (ABC)^23^, Beck Anxiety Inventory (BAI)^24^, Headache Impact Test (HIT-6)^25^, Niigata Persistent Postural-Perceptual Dizziness (PPPD)^26^, and Dizziness Handicap Inventory (DHI)^27^ questionnaires, respectively. Further, the pathological ability to hear individuals’ own voice or internal bodily sounds was measured in 8 participants with SCDS and 7 healthy controls using the Autophony Index questionnaire^6^; this was either administrated directly by our research team or obtained via medical records of individuals with SCDS, if concurrently completed by their medical team.

### Kinematic measurements

**Fig. 1:**
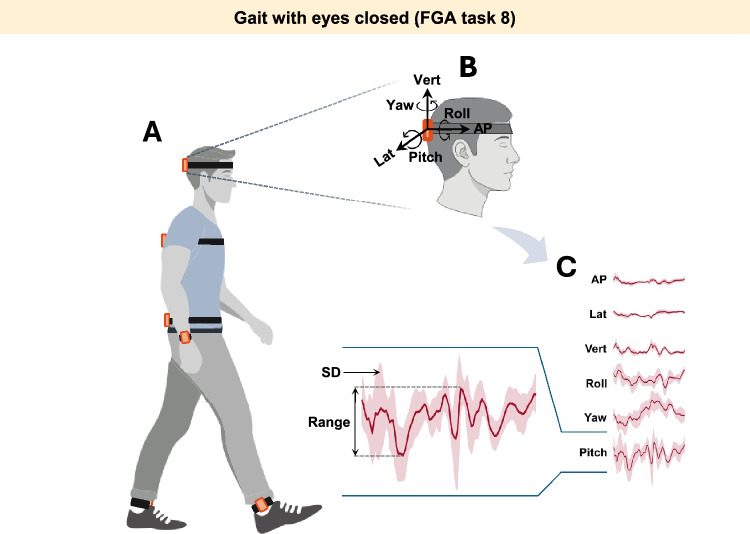
Kinematic measurement setup. (**A**) IMUs were affixed on the head, back, waist, dominant wrist, and ipsi-lesional and contra-lesional ankles. (**B**) Demonstrating 3D axes along which the linear accelerations (i.e., antero-posterior, lateral, and vertical) and angular velocities (i.e., yaw, roll, and pitch) were recorded. (**C**) Raw data of the head movement of a typical participant with SCDS, over a gait cycle while walking with eyes closed (FGA 8). The images in figures A,B and insets of Fig. 6 were hand-drawn using Adobe Illustrator and Adobe Fresco (version 2025, Adobe Inc., https://www.adobe.com/products/illustrator.html and https://www.adobe.com/ca/products/fresco.html. No third-party content was used or modified).

Six inertial measurement units (IMUs; Shimmer3, Shimmer Research, Dublin, Ireland) were attached to the back of participants’ head (head IMU), back of upper trunk at the level of T6-T7 vertebrae (back IMU), lower back approximately between L4-S1 vertebrae (waist IMU), distal of their dominant forearm immediately above and dorsal of the wrist joint (wrist IMU), and on distal-lateral aspect of both shanks immediately above the lateral malleoli (ankle IMUs) (Fig. 1A). Then, participants were asked to perform 10 gait tasks of the FGA scale (Table 3). To avoid falls and to ensure safety during the tests, researchers stood at participants’ side and walked with individuals with SCDS during the gait tasks; additionally, rest breaks were provided as needed during the assessment sessions.

**Table 3:** List of the tasks of Functional Gait Assessment Scale (FGA).

Tasks	Description
FGA 1	Gait on level surface
FGA 2	Gait with change in speed
FGA 3	Gait with horizontal head turns
FGA 4	Gait with vertical head turns
FGA 5	Gait and pivot turn
FGA 6	Gait and stepping over obstacle
FGA 7	Gait with narrow base of support
FGA 8	Gait with eyes closed
FGA 9	Ambulating backwards
FGA 10	Steps

IMUs were synchronized at the beginning of each session using the ConsensysPro software (Shimmer Research, Dublin, Ireland). Each IMU recorded triaxial linear accelerations along the antero-posterior, mediolateral, and vertical axes (set at ± 8 g), as well as triaxial angular velocities in the yaw, pitch, and roll planes (set at ± 2000 deg/s) (Fig. 1B-C). Signals were sampled at 500 Hz and saved on the built-in micro-SD cards for offline processing.

Gait cycles (strides) were defined by successive initial contacts of the same foot—heel strikes for forward walking and toe strikes for backward walking. Initial contacts were automatically detected using a custom MATLAB script based on template matching. Experts first labeled initial contacts in a subset of healthy participants; the corresponding IMU signals were z-score normalized, segmented, and averaged to create a template. For each new participant, z-score normalized IMU signals were compared to this template using a sliding-window cosine similarity metric. A fixed threshold was applied to identify likely initial contacts, which were then reviewed and curated by experts to ensure accurate segmentation. Gait cycles with unclear start or end contacts were excluded from analysis. Then, ipsi-lesional gait cycles for the SCDS group, and right gait cycles for the healthy controls were used to segment all time-series recorded by their remaining IMUs. Data was filtered at 25 Hz using a Butterworth zero-shift 4th order low-pass filter.

We next time-normalized each gait cycle from ipsi-lesional ankle initial contact (0%) to the subsequent ipsi-lesional ankle initial contact (100%) by linearly interpolating the time series to a 0–100% scale. Then, for each participant we computed (1) the average range of linear accelerations and angular velocities across cycles, (2) the standard deviation (SD) of these signals across cycles, as well as (3) their cycle-to-cycle variability, defined as the SD divided by the range for each kinematic measure. Additionally, for each task, average gait cycle duration and its SD and coefficient of variation (CV; measured as the SD divided by the mean) were calculated. To obtain the step length and step time asymmetry measures, we first computed the integration of the upward vertical head accelerations, and the time intervals for each side of movement separately, and then divided these values for the ipsi-lesional steps by the contra-lesional steps, respectively^28,29^.

Additionally, we estimated gait speed for all FGA tasks using ankle IMU data: specifically, we integrated body-frame accelerations from each ankle IMU to obtain global-frame velocity, applying a zero-velocity update during stance to correct for integration drift. Average gait speed was then computed over segmented gait cycles and averaged between ankles. For FGA tasks 1 (gait on level surface) and 8 (gait with eyes closed)—the only tasks where this was possible—we also calculated speed by dividing distance by time. These ground-truth values were used both to confirm our IMU-based estimates and in subsequent analyses.

Finally, we computed global kinematic scores to evaluate whether we could use a reduced set of gait tasks and/or a single IMU to effectively distinguish between the healthy controls and SCDS groups. Global kinematic scores were computed as follows: (i) the calculated kinematic measures (e.g., range of motion, cycle to cycle variability, etc. in each axis) during each task was linearly normalized from mean ± 2SD to a number between 0 and 100 (i.e., normalized mean = 50 and normalized SD = 25); (ii) outliers were projected to either upper bound (100, normal) or lower bound (0, most severely impaired); and (iii) the normalized numbers across gait tasks were then averaged^19,29^. To then identify the most informative gait task subset and IMU placement, we conducted an a-priori optimization approach (Supplementary Fig. 1). Specifically, for each combination of FGA tasks and IMU placement, we derived the probability distribution of kinematic scores based on range of motion in all axes from the healthy controls and SCDS groups and quantified the magnitude of between-group dissimilarity with the minimal transportation cost (also known as Earth Mover’s Distance index, EMD)^30^. The top three gait tasks with the highest EMD were then identified for each single IMU, and the most frequently appearing FGA tasks across all IMUs were considered as the most informative and optimal gait task subset. The optimal global kinematic scores were finally computed from all kinematic measures of the optimal gait task subset for each IMU. A total global kinematic score based on all IMUs and all gait tasks was also computed as a reference to evaluate the performance of our optimization approach. In addition, similar global scores were computed based on the same approach using gait speed alone to assess whether gait speed could distinguish healthy individuals from those with SCDS.

### Statistical analysis

Comparisons between healthy controls and the unilateral SCDS group were performed using a non-parametric independent permutation test (re-randomization) with 100,000 randomized rearrangements of data points. This test is robust for small sample sizes and does not rely on assumptions of normality^31^. In addition, to calculate the correlations between the kinematic and clinical measures of individuals with unilateral SCDS, normality of the data was first examined by using the Shapiro–Wilk test; then, the corresponding Spearman’s or Pearson’s correlation coefficients were computed. Accordingly, to find the consistent correlation trends among gait tasks and clinical measures, we assessed whether the correlations for the most gait tasks: (a) were statistically significant (*p* < 0.05), and (b) had the same sign (i.e., correlations were consistently positive/negative across multiple tasks). A correction for multiple comparisons was not applied to avoid inflating Type II error, given the exploratory nature of the analysis, small sample size, and intent to identify potential associations between kinematics and clinical measures. All statistical analysis and data processing were performed using custom written MATLAB codes (2021b, The MathWorks Inc., Natick, MA, USA); statistical significance level was set at α = 0.05, and values reported as mean ± 1SD.

## Results

**Fig. 2:**
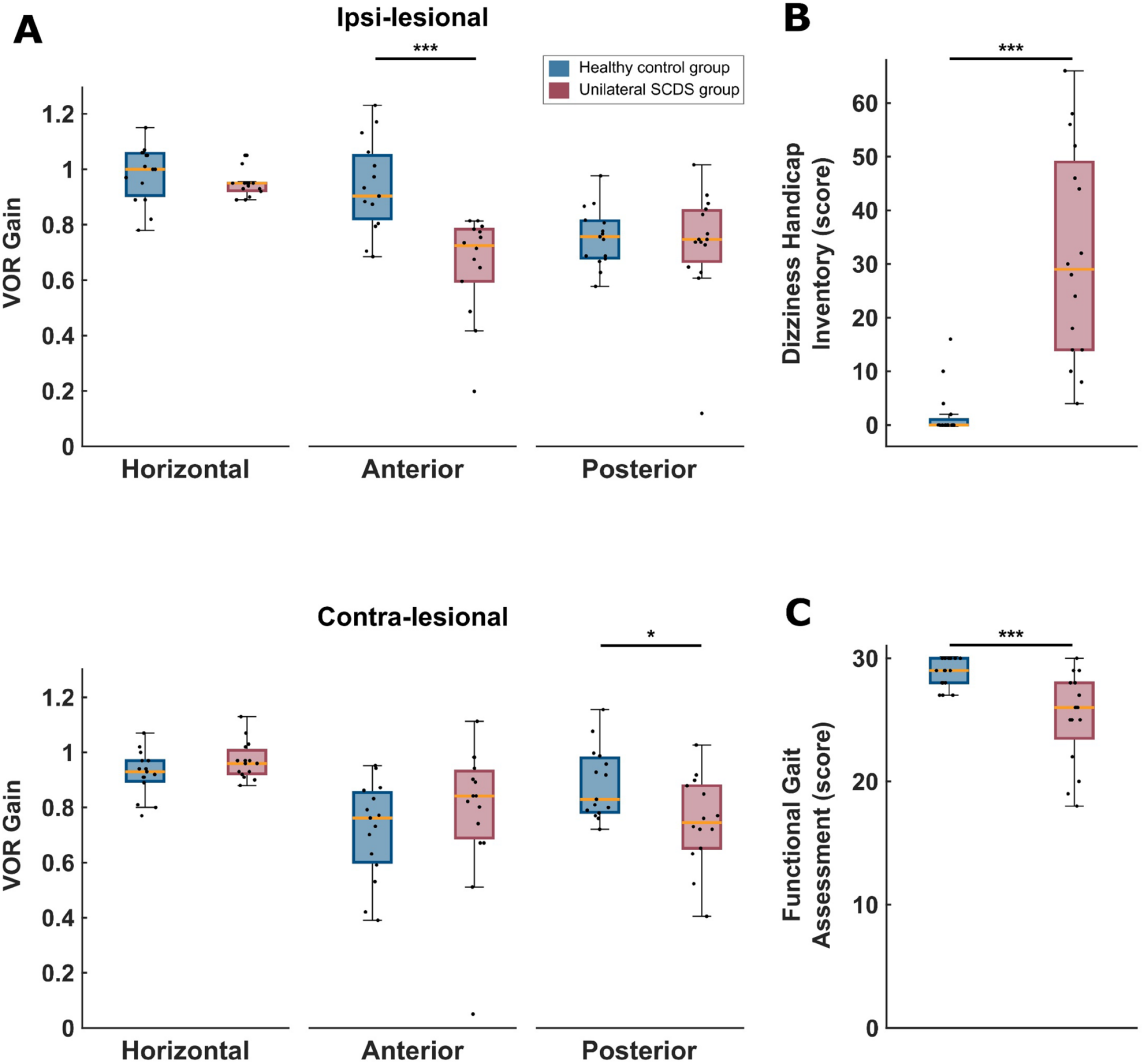
Comparison of clinical measures between the healthy controls (blue) and unilateral SCDS group (red). Black dots represent the individual data points, and orange lines represent the median. (**A**) Between-group differences of the vHIT-induced VOR gains recorded from the ipsi-lesional semicircular canals of SCDS group vs right semicircular canals of healthy controls (top), and contra-lesional semicircular canals of SCDS group vs left semicircular canals of healthy controls (bottom). (**B**) Between-group differences of Dizziness Handicap Inventory score, and (**C**) total score of the Functional Gait Assessment scale. * *p* < 0.05, and *** *p* < 0.001.

To first assess semicircular canal function in our participants with unilateral SCDS, we measured the vestibulo-ocular reflex (VOR) using the video Head Impulse Test (vHIT) and compared their gains to those of healthy controls (Fig. 2A). Participants with unilateral SCDS displayed significantly reduced VOR gains in the ipsi-lesional anterior canal (*p* < 0.001) and the contra-lesional posterior canal (*p* < 0.05). In contrast, VOR gains in the remaining semicircular canals did not differ significantly between groups (*p* > 0.05). Additionally, participants with SCDS reported a significantly greater impact of dizziness on daily life activities (Fig. 2B, *p *< 0.001) and demonstrated significantly lower total scores on the Functional Gait Assessment (FGA) compared to controls (Fig. 2C , *p* < 0.001). Further clinical testing (Table 1) also revealed that the SCDS group walked at a slower self-selected speed, took longer to complete both single-task and cognitive-dual task Timed Up and Go (TUG) tests, and reported lower balance confidence (*p* < 0.01). These individuals also exhibited elevated levels of anxiety, oscillopsia, and autophony (all *p* < 0.01).

### Pitch velocity and vertical acceleration measures demonstrate gait differences between healthy controls and individuals with unilateral SCDS

**Fig. 3:**
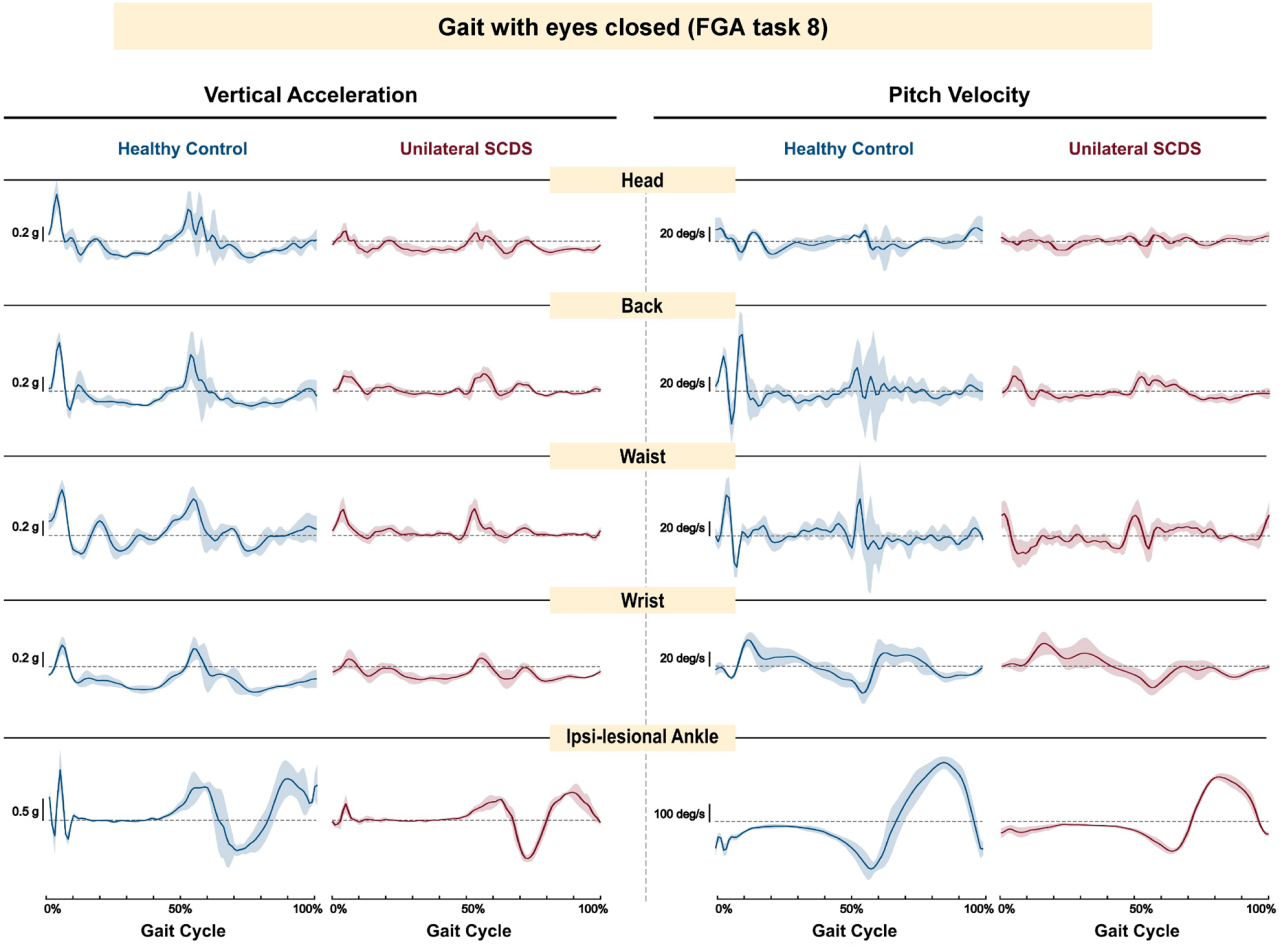
Examples of raw vertical accelerations (left panels), and raw pitch velocities (right panels) extracted from head, back, waist, dominant wrist, and ipsi-lesional ankle (top to bottom) from a typical healthy control male participant (blue traces) and a typical male participant with SCDS (red traces) during walking with eyes closed (FGA 8).

Figure 3 shows representative data recorded over an average gait cycle, illustrating vertical acceleration (left) and pitch velocity (right) signals for a typical healthy control (blue traces) and a unilateral SCDS participant (red traces). The signals shown were recorded from the IMUs located on the head, back, dominant wrist, waist, and ipsi-lesional ankle (top to bottom) while walking with eyes closed (FGA task 8). Compared to the control participant, the individual with SCDS showed reduced pitch velocity and vertical acceleration throughout the gait cycle. This individual also demonstrated reduced VOR gains on the ipsi-lesional side, 0.68 (anterior) and 0.75 (posterior), relative to 0.89 on the contra-lesional side for both anterior and posterior canal testing.

**Fig. 4:**
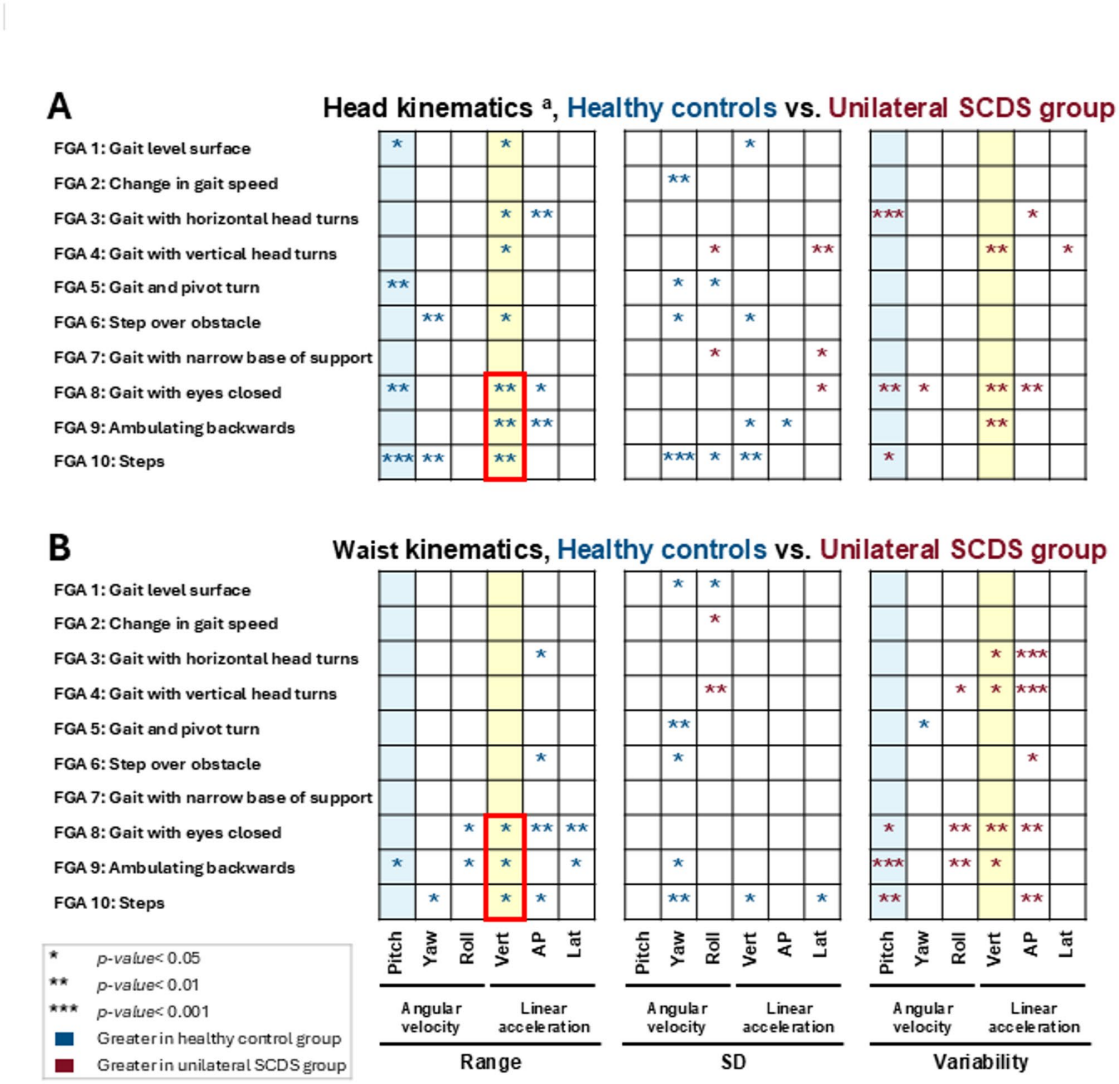
Between-group comparisons of angular velocities (pitch, yaw, roll) and linear accelerations (vertical, antero-posterior, lateral) of (**A**) head, and (**B**) waist during all 10 tasks of the Functional Gait Assessment (FGA) scale. Blue asterisks indicate statistically significant differences with greater values for the healthy control group, and red asterisks indicate statistically significant differences with greater values for the SCDS group. Light blue columns are range of pitch velocity and variability of pitch velocity; light yellow columns are range of vertical acceleration and variability of vertical acceleration. Red boxes highlight the gait tasks consistently showing a reduced range of vertical acceleration in the SCDS group. *p < 0.05, **p < 0.01, and ***p < 0.001. SD: standard deviation. The corresponding numerical values (mean ± SD) of each kinematic measure for both healthy control and SCDS groups have been presented in Supplementary Tables 1–3. ^**a**^: Head IMU data was available for 15 individuals with SCDS and 16 healthy controls.

Between-group comparisons are shown in Fig. 4 for all 10 gait tasks of the Functional Gait Assessment scale (FGA). Figure 4A illustrates comparisons of the ranges, standard deviations (SD), and cycle-to-cycle variabilities of the linear acceleration (i.e., vertical, antero-posterior, lateral) and the angular velocity (i.e., pitch, yaw, roll) of the motion signals recorded from the head-mounted IMU. Figure 4B illustrates the corresponding between-group comparisons for the motion signals recorded from the waist-mounted IMU. The corresponding mean ± SD values for motion signals recorded by both IMUs are provided in Supplementary Tables 1–3.

Overall, our analysis of head motion kinematics revealed that individuals with unilateral SCDS exhibited reduced ranges of angular velocity and linear acceleration during gait compared to controls (Fig. 4A). Notably, the range of pitch head velocity was significantly lower in the SCDS group during four gait tasks: level surface walking, gait with pivot turn, walking with eyes closed, and stair negotiation (*p* < 0.05). Similarly, the range of vertical head acceleration was significantly reduced in seven out of ten tasks (*p* < 0.05), with the exception of walking with change in speed, gait with pivot turn, and walking with a narrow base of support. In addition to these reductions in ranges, the SCDS group exhibited greater variability of pitch head velocity while walking with horizontal head turns, walking with eyes closed, and stair negotiation (*p* < 0.05). Similarly, a significantly greater variability in vertical head acceleration was observed in the SCDS group during gait with vertical head turns, gait with eyes closed, and ambulating backwards (*p* < 0.01).

Our parallel quantitative analysis of waist kinematics (Fig. 4B) revealed a similar pattern. The range of vertical acceleration generated was generally lower in the SCDS group, reaching significance while walking with eyes closed, ambulating backwards, and stair negotiation (*p* < 0.05). Furthermore, the reduction in the range of vertical acceleration was accompanied by significantly greater vertical acceleration variability during gait with horizontal head turns, gait with vertical head turns, gait with eyes closed, and ambulating backwards (*p* < 0.05). Additionally, compared to controls, pitch waist velocity range was lower for the SCDS group during the ambulating backwards task (*p* < 0.05), and also displayed significantly increased variability during this task, as well as during gait with eyes closed, and stair negotiation (*p* < 0.05).

**Fig. 5:**
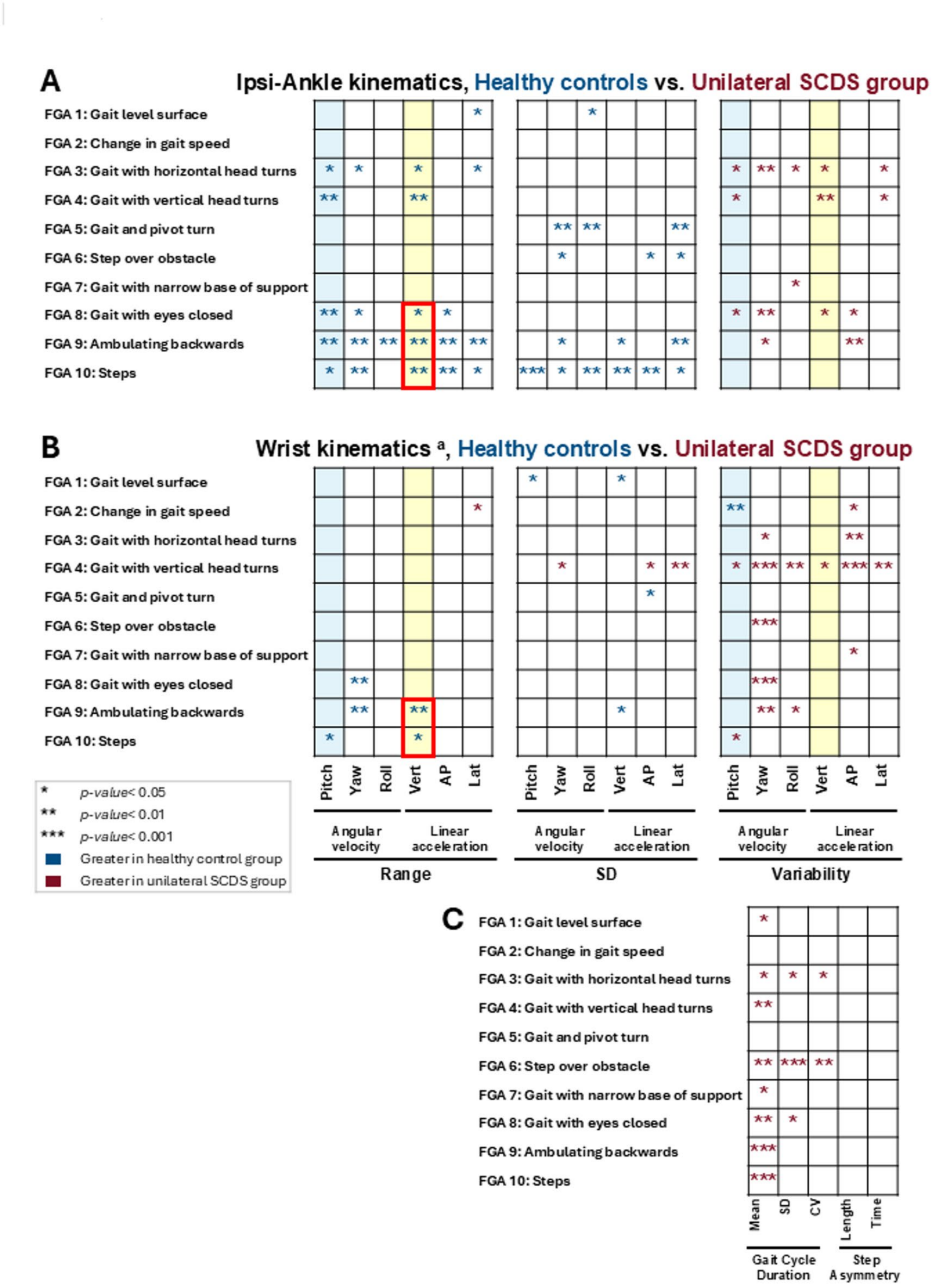
Between-group comparisons of angular velocities (pitch, yaw, roll) and linear accelerations (vertical, antero-posterior, lateral) of the (**A**) ipsi-lesional ankle, and (**B**) dominant wrist during all 10 tasks of the Functional Gait Assessment (FGA) scale. Blue asterisks indicate statistically significant differences with greater values for the healthy control group, and red asterisks indicate statistically significant differences with greater values for the SCDS group. Ipsi-lesional ankle: Denotes the symptomatic side for SCDS group, and right side for control group. Light blue columns are range of pitch velocity and variability of pitch velocity; light yellow columns are range of vertical acceleration and variability of vertical acceleration. Red boxes highlight the gait tasks consistently showing a reduced range of vertical acceleration in the SCDS group. The corresponding numerical values (mean ± SD) of each kinematic measure for both healthy control and SCDS groups have been presented in Supplementary Tables 4–6. (**C**) Between-group differences of the mean, standard deviation (SD), and coefficient of variation (CV) of the gait cycle durations, and time and step length asymmetries during each task of the FGA scale. The corresponding numerical values (mean ± SD) for both healthy control and SCDS groups have been presented in Supplementary Table 7. *p < 0.05, **p < 0.01, and ***p < 0.001. ^**a**^: Wrist IMU data was available for 16 individuals with SCDS and 15 healthy controls.

Figure 5 presents the same kinematic analyses shown in Fig. 4—range, standard deviation, and cycle-to-cycle variability of linear acceleration and angular velocity—now applied to motion signals recorded from the ipsi-lesional ankle (Fig. 5A) and dominant wrist (Fig. 5B) during the 10 FGA tasks. The corresponding mean ± SD values are provided in Supplementary Tables 4–6. As described above for head and waist motion, individuals with unilateral SCDS generally showed reduced range and increased variability for ankle and wrist motion compared to controls. Specifically, pitch velocity range was significantly lower in the SCDS group at the ankle during five gait tasks and at the wrist during stair negotiation (*p* < 0.05). In SCDS group, variability in pitch velocity was significantly greater at the ankle during tasks involving head turns, as well as while walking with eyes closed (*p* < 0.05). At the wrist, pitch velocity variability in the SCDS group was significantly greater than in healthy controls during walking with vertical head turns and stair negotiation (*p* < 0.05), but significantly lower during walking with changes in speed (*p* < 0.01).

Correspondingly, vertical acceleration range at the ankle was also significantly reduced in SCDS during five gait tasks (*p* < 0.05). Variability in vertical acceleration at ankle was significantly greater in SCDS in gait tasks with head turns, and walking with eyes closed (*p* < 0.05). Variability in vertical acceleration of wrist was significantly greater in SCDS group while walking with vertical head turns (*p* < 0.05). Figure 5C further shows that gait cycle duration was generally longer in the SCDS group across 8 of 10 tasks (*p* < 0.05), indicating slower stride timing. The SCDS group also exhibited greater variability in gait cycle duration (SD and CV) during tasks with head turns, eyes closed, and obstacle negotiation (*p* < 0.05). Mean ± SD values for gait cycle duration across tasks are provided in Supplementary Table 7. No group differences were observed in step length or step time asymmetry (*p* > 0.05).

Thus, in summary, individuals with unilateral SCDS exhibited reduced pitch velocity and vertical acceleration ranges, increased variability in pitch velocity and vertical acceleration, and slower, more variable gait cycle timing compared to healthy controls. These differences were most evident during tasks that challenged balance, such as walking with head turns, walking with eyes closed, and obstacle negotiation. Despite these impairments, step length and step time symmetry remained intact. Similar results were observed when the same kinematic analyses were applied to the back and contra-lesional ankle IMUs (see Supplementary Fig. 2 and Supplementary Tables 8–10).

Finally, to determine whether the reduced head pitch velocity and vertical acceleration observed in individuals with SCDS could simply be explained by their slower gait, we analyzed the relationship between gait speed and head kinematics in healthy controls (Table 4; see Supplementary Table 7 for gait speeds across tasks) to determine whether this association is normally present. Vertical head acceleration was significantly correlated with gait speed in 6 of 7 tasks (*p* < 0.05), with the exception of stair negotiation (FGA task 10), while backward walking (FGA task 9) was only marginally correlated. In contrast, head pitch velocity showed a significant correlation with gait speed in only one task—level-ground walking (FGA task 1)—and no correlation in tasks 5, 8, or 10 (*p* > 0.05). These findings suggest that while slower gait speed may contribute to some of the kinematic alterations observed in SCDS, it does not fully account for them—particularly the changes in head pitch velocity—indicating that individuals with SCDS adopt additional protective gait strategies beyond simply walking more slowly.

**Table 4:** Correlations between task-specific gait speeds and corresponding vertical head acceleration and pitch velocity during the FGA tasks with significant differences between healthy controls and individuals with unilateral SCDS.

	Healthy control group		Unilateral SCDS group	
	correlation coeff	*p* value	correlation coeff	*p* value
Correlation between range of head vertical acceleration and task-specific gait speeds				
FGA 1	0.644	0.0071	0.671	0.0062
FGA 3	0.709	0.0021	0.784	0.0006
FGA 4	0.763	0.0006	0.773	0.0007
FGA 6	0.708	0.0022	0.779	0.0010
FGA 8	0.850	0.00003	0.748	0.0013
FGA 9	0.506	0.0479	0.596	0.0213
FGA 10	− 0.223	0.4060	0.501	0.0572
Correlation between range of head pitch velocity and task-specific gait speeds				
FGA 1	0.553	0.0264	− 0.022	0.9390
FGA 5	0.140	0.6060	0.242	0.3850
FGA 8	0.180	0.5040	0.484	0.0678
FGA 10	0.047	0.8620	– 0.134	0.6340

FGA, Functional gait assessment scale; SCDS, superior canal dehiscence syndrome; FGA1, Gait on level surface; FGA2, Gait with change in speed; FGA3, Gait with horizontal head turns; FGA4, Gait with vertical head turns; FGA5, Gait and pivot turn; FGA6, Gait and stepping over obstacle; FGA7, Gait with narrow base of support; FGA8, Gait with eyes closed; FGA9, Ambulating backwards; FGA10: Steps. coeff.: Coefficient. Significance level α = 0.05.

### Global kinematic scores derived from targeted IMU placements and gait tasks accurately differentiate individuals with SCDS

**Fig. 6:**
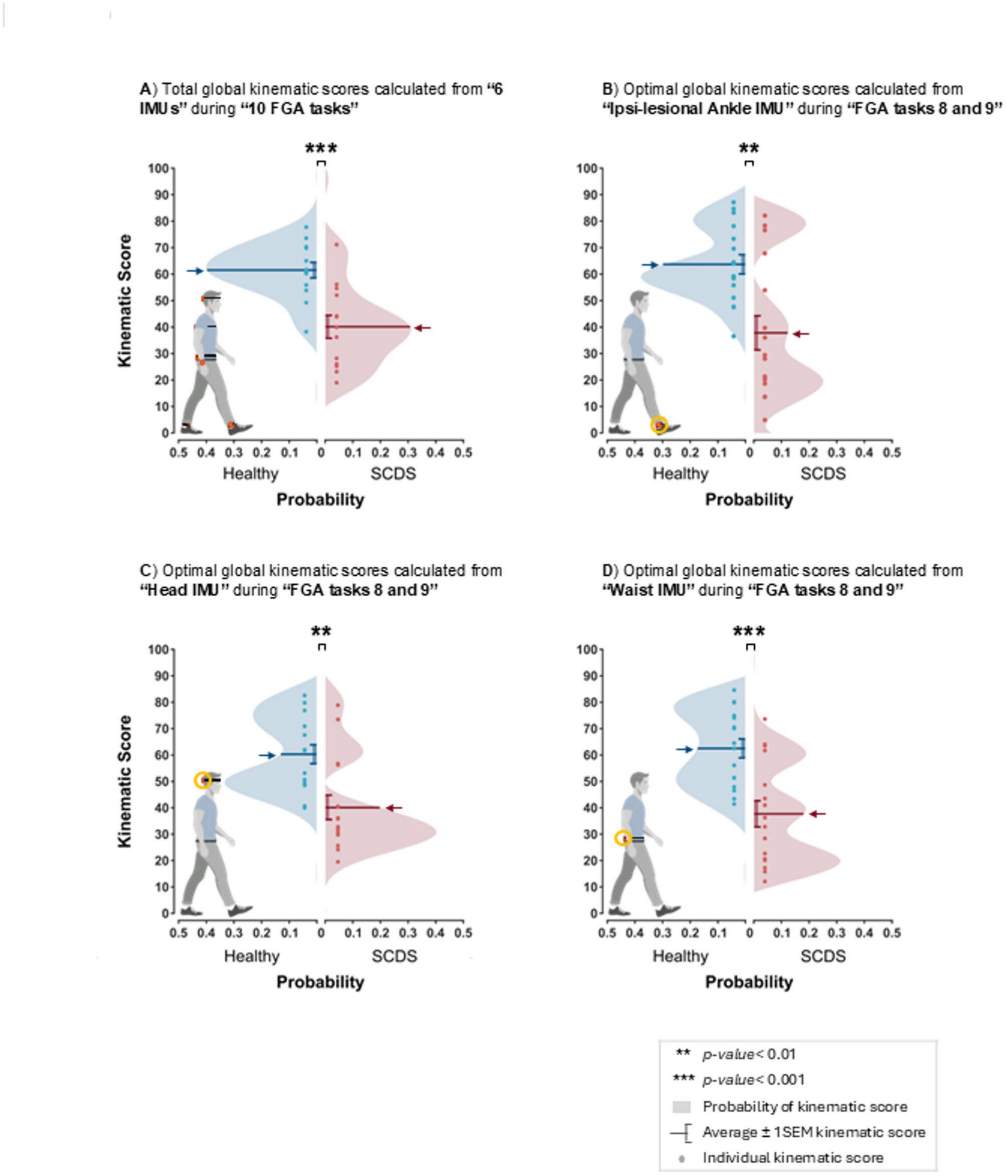
Differentiating between the healthy control (blue) and SCDS (red) groups using four global kinematic scores: (**A**) the total global kinematic score derived from all 6 IMUs and all 10 tasks, using all kinematic measures; (**B**) global kinematic score derived from the Ipsi-lesional ankle IMU, from combination of FGA8 and FGA9, and using all kinematic measures; (**C**) global kinematic score derived from Head IMU, from combination of FGA8 and FGA9, using all kinematic measures; and (**D**) global kinematic score derived from the Waist IMU, from combination of FGA8 and FGA9, using all kinematic measures. Arrows represent the average global kinematic score for each group, and vertical bars represent ± 1SEM. The shaded areas represent the probability distribution of the global kinematic scores for each group. **p < 0.01, ***p < 0.001. The corresponding numerical values of these global kinematic scores have been presented in Supplementary Table 12.

Given the need for practical clinical tools, we next assessed whether global kinematic scores derived from targeted IMU placements and specific gait tasks could reliably differentiate individuals with SCDS from healthy controls. First, a total global kinematic score was computed using the data from all IMUs, and all linear and angular kinematic measures during all FGA tasks, which then compared between the healthy and SCDS groups (Fig. 6A). Next, an a-priori analysis was conducted to identify the most informative subset of the FGA tasks (see Methods). Supplementary Table 11 presents the values of individual global kinematic scores calculated from the individual IMUs for each FGA task. This analysis revealed that ambulating backwards (FGA task 9), walking with eyes closed (FGA task 8), and stair negotiation (FGA task 10) were the most informative gait tasks for demonstrating differences between healthy controls and individuals with SCDS. Finally, we calculated global kinematic scores for each IMU using the most informative gait tasks identified above. Supplementary Table 12 presents the values of the global kinematic score for each of the 6 IMUs, calculated from all linear and angular kinematic measures, using the combination of FGA tasks 8 and 9. (Note that since the safe evaluation of stair negotiation (FGA task 10) may not be possible in routine clinical settings, global kinematic scores incorporating FGA task10 are not presented but their values are available in Supplementary Table 13). Overall, these results demonstrate that IMUs placed on the head, waist, and ankles provided better differentiation between individuals with SCDS and healthy controls than IMUs placed on the back or wrist.

Finally, we compared the optimal global kinematic scores between healthy controls and individuals with SCDS based on data from the ipsi-lesional ankle (Fig. 6B), the head (Fig. 6C), and the waist (Fig. 6D) during FGA tasks 8 and 9. Optimal global kinematic scores calculated from the contra-lesional ankle performed nearly equally to those of the ipsi-lesional ankle (available in Supplementary Tables 12 and 13). Overall, all four types of global kinematic scores (Fig. 6A–D) consistently showed that the healthy control group scored higher than the SCDS group. Moreover, the global kinematic scores derived from a single IMU and a reduced set of gait tasks effectively distinguished individuals with SCDS from healthy controls (*p* < 0.01) and demonstrated comparable discriminative ability to the total global kinematic score calculated using all 10 tasks and all IMUs. Thus, taken together these findings suggest that, for clinical applications, using a single IMU placed on the head, waist, or ipsi-lesional ankle (or alternatively, on contra-lesional ankle) during gait with eyes closed and ambulating backwards may be sufficient to differentiate individuals with SCDS from healthy controls.

Finally, to evaluate whether gait speed alone could distinguish individuals with SCDS from healthy controls as effectively as the global kinematic scores derived from individual IMUs, we computed three gait speed-based scores using the same analytical approach. These included: (1) normal gait speed over a 10-m walkway, (2) a combined score from FGA tasks 8 and 9, and (3) a combined score from FGA tasks 8, 9, and 10. All three gait speed scores significantly differentiated between groups, but with lower or comparable statistical significance than the global kinematic scores from the ankle, waist, or head IMUs. Additionally, the gait speed scores based on FGA tasks 8–9 and 8–10 showed smaller Earth Mover’s Distance (EMD) values than the ankle- and waist-based kinematic scores, indicating greater overlap between groups. The 10-m walk score yielded the smallest group separation, with an EMD generally lower than all IMU-based scores (see Supplementary Table 14). These findings suggest that although gait speed captures some between-group differences, it lacks the discriminative power of full-body kinematic measures derived from wearable sensors.

### Correlations between gait kinematics and clinical outcomes in individuals with SCDS

**Fig. 7:**
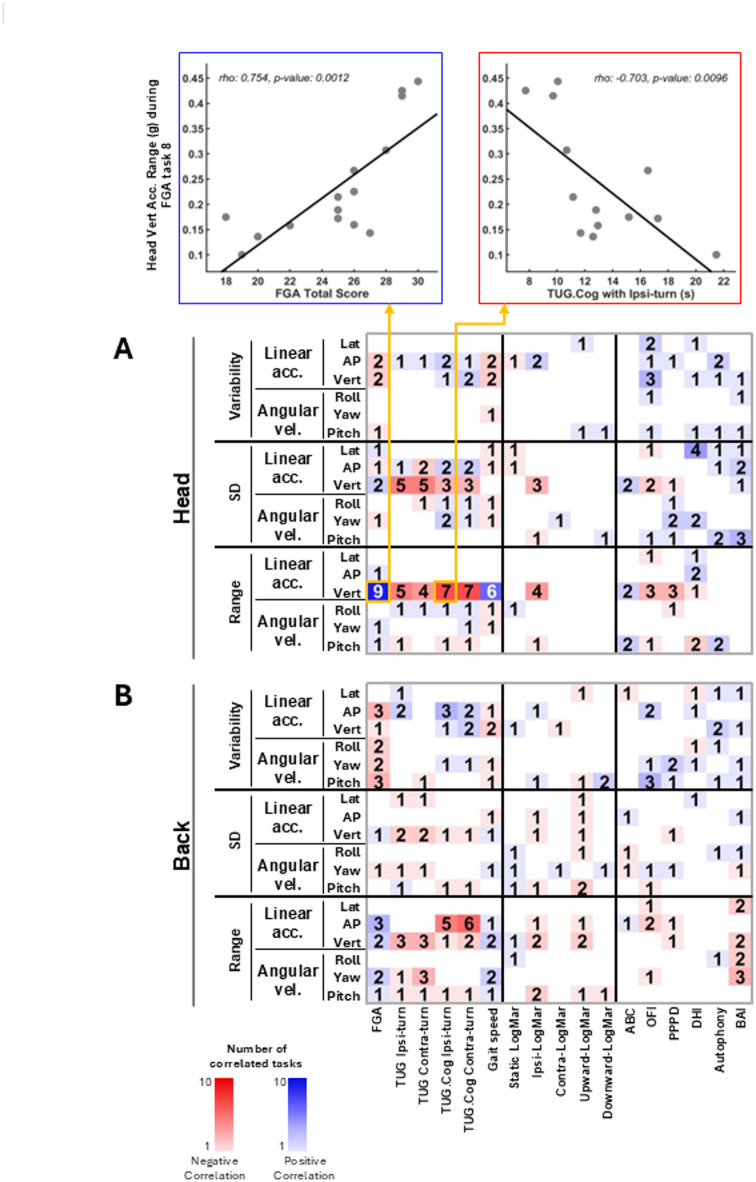
Maps of correlations among the clinical measures of individuals with SCDS (x-axis) and their kinematic gait measures (y-axis), calculated for: (**A**) head IMU, and (**B**) back IMU. Blue and red squares reflect positive and negative correlations, respectively. Brightness and the numbers in each cell of the matrix indicate the number of tasks (out of 10) in which there was a significant correlation between the kinematic and clinical measures (*p* < 0.05). The two top scatter plots illustrate examples of correlations between the range of the head vertical acceleration during walking with eyes closed and the total score of FGA (significant positive correlation; left plot); and between the range of the head vertical acceleration during walking with eyes closed and the time of cognitive TUG test (significant negative correlation; right plot). SD: standard deviation. acc: acceleration. vel: velocity. Ipsi: ipsilesional. Contra: contralesional. FGA: Functional Gait Assessment scale. TUG: Timed Up and Go test. TUG.Cog: cognitive (dual task) Timed Up and Go test. Lat: lateral. Vert: vertical. AP: antero-posterior. OFI: Oscillopsia Functional Impact questionnaire. DHI: Dizziness Handicap Inventory questionnaire. BAI: Beck Anxiety Index questionnaire. PPPD: Niigata Persistent Postural-Perceptual Dizziness questionnaire. DHI: Dizziness Handicap Inventory questionnaire. Autophony: Autophony Index questionnaire. ABC: Activities-specific Balance Confidence questionnaire. Kinematic data of the head IMU was available for 15 individuals with SCDS.

**Fig. 8:**
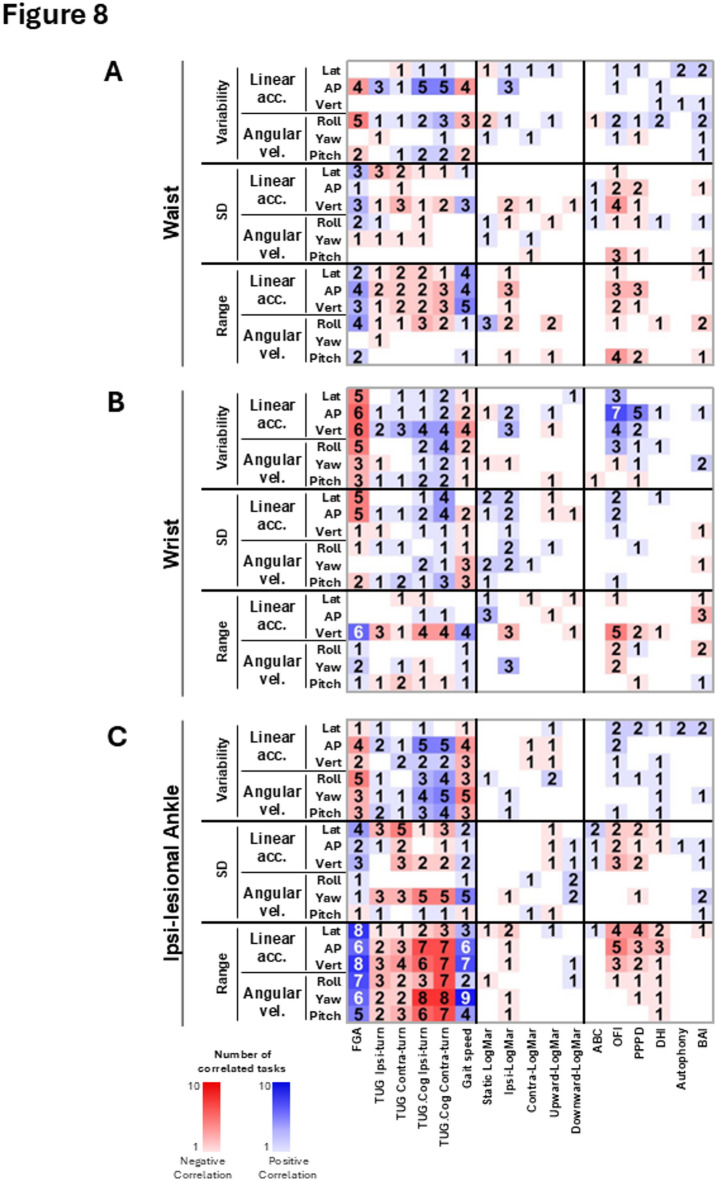
Maps of correlations among the clinical measures of individuals with SCDS (x-axis) and their kinematic gait measures (y-axis) calculated for: (**A**) waist IMU, (**B**) dominant wrist IMU, and (**C**) ipsi-lesional ankle IMU. Blue and red squares reflect positive and negative correlations, respectively. Brightness and the numbers in each cell of the matrix indicate the number of tasks (out of 10) in which there was a significant correlation between the kinematic and clinical measures (*p* < 0.05). SD: standard deviation. acc: acceleration. vel: velocity. Ipsi: ipsilesional. Contra: contralesional. FGA: Functional Gait Assessment scale. TUG: Timed Up and Go test. TUG.Cog: cognitive (dual task) Timed Up and Go test. Lat: lateral. Vert: vertical. AP: antero-posterior. OFI: Oscillopsia Functional Impact questionnaire. DHI: Dizziness Handicap Inventory questionnaire. BAI: Beck Anxiety Index questionnaire. PPPD: Niigata Persistent Postural-Perceptual Dizziness questionnaire. DHI: Dizziness Handicap Inventory questionnaire. Autophony: Autophony Index questionnaire. ABC: Activities-specific Balance Confidence questionnaire.

Figure 7A, B illustrates the correlations between linear and angular gait kinematics of individuals with SCDS, recorded by the IMUs placed on the head and back, and their functional gait measures, functional vestibular measures, and participant-reported outcome measures (PROMs). Figures 8A–C demonstrate the correlations between the kinematic measures of the waist, dominant wrist, and ipsi-lesional ankle and SCDS group’s functional gait measures, functional vestibular measures, and PROMs. In both figures, the numbers in each cell represent the number of gait tasks (out of 10) for which a specific kinematic measure was significantly correlated with a specific clinical measure (*p* < 0.05). Supplementary Fig. 3 represents the same correlational analysis for the kinematic measures of the contra-lesional ankle of the SCDS group. Across all IMUs in the abovementioned figures, the most consistent significant correlations were observed between the kinematic measures and the functional gait measures—most notably with FGA total score, cognitive-dual task TUG, and gait speed. One of the strongest and most consistent correlations was between the FGA total score and the range of head vertical acceleration (*p* < 0.05 in 9 out of 10 gait tasks; Fig. 7A), suggesting that a greater range of head vertical acceleration is associated with better gait function in individuals with unilateral SCDS.

In contrast, fewer significant correlations were found between the gait kinematic measures and participant-reported measures, and to a lesser extent, with functional vestibular measures. Among the functional vestibular measures, one of the most consistent correlations was between the range of head vertical acceleration and the ipsi-lesional yaw LogMAR score (*p* < 0.05 in 4 out of 10 gait tasks; Fig. 7A) indicating that a greater range of head vertical acceleration during gait is associated with better ipsi-lesional visual acuity (i.e., a smaller LogMAR score). Among the PROMs, one of the strongest and most consistent correlations was observed between the variability of antero-posterior acceleration of the dominant wrist and the Oscillopsia Functional Index (OFI) score (*p* < 0.05 in 7 out of 10 gait tasks; Fig. 8B). This finding suggests that individuals with SCDS who experience more severe “bouncy vision” during their daily activities tend to walk with more variability in acceleration of their arm swing antero-posteriorly. Thus, taken together, these findings highlight that objective gait kinematics, particularly head and upper limb dynamics, are closely linked to functional gait performance in individuals with SCDS, while showing more limited associations with vestibular function and self-reported symptoms.

## Discussion

To our knowledge, this study is the first to objectively quantify head and body kinematics during locomotion in individuals with superior canal dehiscence syndrome (SCDS). Here we investigated the effects of unilateral SCDS on the movement kinematics while participants completed a series of walking tasks with varying levels of challenge as defined by the Functional Gait Assessment (FGA) scale. We recorded triaxial linear accelerations (antero-posterior, vertical, and lateral) and triaxial angular velocities (pitch, roll, and yaw) from IMUs placed on the head, back, waist, dominant wrist, and both ankles. Overall, our findings indicated that individuals with unilateral SCDS not only exhibit impaired gait function compared to healthy controls but also show distinct alterations in head and body kinematics during gait. Specifically, individuals with unilateral SCDS demonstrate restricted but more variable pitch velocity and vertical acceleration, particularly in their head motion. These differences become more pronounced for gait tasks with increased sensory and motor challenge, such as walking with eyes closed, stair negotiation, walking backwards, and walking while rotating the head.

The diagnosis of SCDS is typically confirmed through high-resolution computed tomography scans of the temporal bone, which reveal evidence of dehiscence, along with characteristic physiological signs and symptoms^32,33^. According to the Bárány Society guidelines, these clinical findings must be consistent with the pathophysiology of the third mobile window syndrome—manifesting as symptoms resulting from a low-impedance pathway for sound or pressure to cause fluid movement in the superior semicircular canal— while excluding other vestibular conditions^4^. A distinguishing feature of SCDS is its axis-specific vestibular effects. Previous research has reported a weak negative correlation between the length of the dehiscence and pre-operative VOR gain for the affected superior canal^13^, and pressure- or sound-induced nystagmus in the plane of the affected canal^34,35^.

One of the key findings of our present study emphasizes these axis-specific effects: individuals with SCDS exhibit a restricted range of vertical acceleration and pitch velocity during walking. This limitation was evident not only in head kinematics but also in the movement patterns of the waist and upper and lower extremities. An exception to this trend was a tendency toward a greater range of pitch velocity recorded by the back IMU in individuals with SCDS compared to controls, which reached statistical significance only during the tandem walking task. We speculate that affected individuals may generate this larger back (upper trunk) pitch velocity as a counteracting mechanism, to ultimately reduce the vertical changes imposed on the head while controlling their balance on a narrow base of support.

Our results further showed that, across all IMUs, individuals with SCDS also generally exhibit greater cycle-to-cycle variability, particularly during walking with vertical or horizontal head turns, walking with eyes closed, and ambulating backwards. Movement variability over multiple repetitions of a task (e.g., taking multiple strides during a gait task) can be considered an indicator of motor exploration required for learning and fine-tuning the performance of an expected task^36,37^. However, we speculate that the effectiveness of this motor exploration process may be diminished in SCDS, as it is potentially interrupted by experiencing disruptive audibility of footsteps and/or oscillopsia during each gait cycle, which in turn leads to increased cycle-to-cycle variability. Future research is needed to examine whether interventions such as gaze stabilization rehabilitation or canal repair surgery can improve gait in SCDS by both reducing cycle-to-cycle variability as well as expanding the restricted range of vertical acceleration and pitch head velocity observed in these individuals.

Previous studies have reported that 50–90% of individuals with SCDS experience unsteadiness^38,39^. However, to date, only one prior study has investigated gait function in pre-surgical individuals with SCDS using subjectively scored Dynamic Gait Index (DGI)^14^. Notably, this prior study reported DGI scores for pre-surgical SCDS individuals that were within the normal range for the healthy population (i.e., 22.8 out of 24). In contrast, our findings demonstrate that individuals with unilateral SCDS exhibit significantly impaired gait performance compared to healthy controls when subjectively scored using the standard Functional Gait Assessment (FGA). This discrepancy may reflect key methodological differences: our study included an age- and sex-matched control group and employed the FGA rather than the DGI. The FGA incorporates three particularly challenging tasks—tandem walking, walking with eyes closed, and ambulating backwards—that may be especially sensitive to the gait deficits associated with SCDS. These results suggest that the DGI may lack the sensitivity required to detect such impairments in this population.

Importantly, a key strength of our study is that we objectively assessed gait alterations during the FGA using a quantitative kinematic approach. The most pronounced group differences—reduced vertical acceleration and pitch head velocity, along with increased cycle-to-cycle variability in individuals with SCDS—emerged during tasks that place high demands on sensory and motor integration, such as walking with eyes closed, ambulating backwards, and stair negotiation. Walking with eyes closed is likely especially challenging for individuals with SCDS, as they cannot rely on visual input and must instead depend on compromised vestibular sensation. Additionally, backward walking requires atypical muscle recruitment patterns compared to forward gait^40^, and stair negotiation demands precise visuomotor coordination to ensure safe foot placement^41^, both of which may exacerbate difficulties for individuals with vestibular dysfunction. Correspondingly, individuals with SCDS walked more slowly than controls, particularly as task demands increased. While slower gait may contribute to reduced head motion, our control-group analysis showed that pitch velocity was largely independent of gait speed—suggesting that the kinematic differences observed in individuals with SCDS reflect more than just walking slower. Taken together, these findings suggest that individuals with SCDS adopt a protective strategy to minimize head motion—particularly in the pitch and vertical axes—in order to reduce stimulation of the superior semicircular canal. Nonetheless, gait speed may play a contributing role and should be accounted for in future studies aiming to disentangle compensatory strategies from speed-related effects.

It is also noteworthy that the changes we observed in the head kinematics due to a single superior canal impairment have some similarities with those observed in unilateral tumoral involvement of the vestibular nerve. For example, a study by Zobeiri et al. (2021) using a comparable approach established that pre-surgical individuals with vestibular schwannoma likewise exhibited a reduced range of vertical head acceleration on half of the FGA tasks when compared to healthy controls^29^. However, the alterations in head kinematics observed in individuals with SCDS were ultimately distinct; in SCDS, the significant reduction in head vertical acceleration and pitch velocity persisted even during normal walking at a comfortable speed (i.e., FGA task 1). This difference may reflect that, in SCDS, gait-related impacts—such as consecutive heel strikes during level walking—can provoke symptoms like autophony and oscillopsia, prompting compensatory reductions in head motion even at normal walking speeds. Such symptom-driven adaptations are less likely in vestibular schwannoma, where symptoms develop more gradually. Our data further suggest that SCDS has a broader functional impact, as individuals appear less able to compensate over time—perhaps due to the sudden, transient nature of the symptom-provoking stimuli. In contrast, the gradual decline in vestibular function with schwannoma may allow more time for neural adaptation. These distinct movement patterns highlight the potential of head kinematics as clinically relevant metrics for assessing impairment and informing prehabilitation strategies.

We further found that individuals reporting more severe oscillopsia exhibited greater variability in anterior–posterior arm swing acceleration across most gait tasks. Arm swing plays a crucial role in gait stability, energy efficiency, and recovery from perturbations^42,43^. In the context of SCDS, increased variability in arm motion may reflect difficulty maintaining interlimb coordination in the presence of destabilizing sensory input, such as blurred vision caused by oscillopsia. Because arm swing is influenced by both central pattern generators and sensory feedback^42^, this variability may also indicate disrupted motor control or compensatory adjustments aimed at maintaining stability. These findings extend the impact of SCDS beyond head kinematics, highlighting arm swing as a clinically relevant and quantifiable indicator of symptom severity and functional disturbance.

Finally, our findings suggest that a global kinematic score derived from a subset of gait tasks using a single IMU (Supplementary Tables 12 and 13) can effectively differentiate individuals with unilateral SCDS from healthy controls. A previous study from our group demonstrated that a global kinematic score—calculated from head kinematic measures during a subset of FGA tasks, including gait with vertical and horizontal head turns, pivot turns, and a narrow base of support—could accurately distinguish individuals with and without vestibular schwannoma^15^. In the present study, we compared global kinematic scores computed using the IMUs that showed the most significant between-group differences (i.e., head, waist, and ankles). Overall, we found that global kinematic scores derived from just two of the most challenging tasks (i.e., FGA tasks 8 and 9), using either the head, waist, or ankle sensors, effectively distinguished SCDS individuals. Scores derived from the waist IMU generally yielded slightly stronger statistical separation between groups. Notably, these single-IMU global kinematic scores performed comparably to the composite score calculated using all IMUs across all tasks. Together, these findings highlight the usefulness of a simplified gait assessment approach, particularly in settings where it is not feasible to use multiple sensors or administer an extensive set of tasks.

While our study establishes that SCDS leads to canal-specific kinematic adaptations during locomotion, several limitations should be considered. We focused on individuals with unilateral symptoms who had not undergone surgical intervention. Therefore, our findings may not be generalizable to individuals with previous surgical interventions or with bilateral SCD symptoms, or individuals with additional neurologic or otologic conditions. Additionally, while our SCDS group had a relatively broad age range (33–68 years), we minimized the potential effects of aging on gait kinematics, by recruiting an age-matched healthy control group.

## Conclusion

Individuals with unilateral SCDS exhibit distinct gait kinematics compared to healthy controls, with the most pronounced alterations—reduced vertical head acceleration, diminished pitch velocity, and increased variability—emerging during tasks that challenge sensory integration and motor coordination, such as walking with eyes closed, ambulating backwards, and stair negotiation. While slower gait may contribute in part to these changes, our findings suggest that reduced head motion likely reflects a protective adaptation aimed at minimizing stimulation of the affected superior semicircular canal. This strategy may help individuals mitigate distressing symptoms such as oscillopsia, dizziness, and the perception of audible footfalls during walking.

## Supplementary Information

Supplementary Information.

## Data Availability

The raw data used in the present study is not publicly available, as the participants in this research did not consent to share their individual data publicly. However, the data may be available from the corresponding author upon reasonable request and approval from the Institutional Review Board.
