# Supplementary material for: Superior canal dehiscence syndrome induces canal-specific kinematic adaptations during locomotion
Source: Sci Rep. 2025 Sep 29;15:33356. doi: 10.1038/s41598-025-16904-7 (PMC12480983; doi:10.1038/s41598-025-16904-7)
Supplement: Supplementary file 1 — Supplementary Information. [file 41598_2025_16904_MOESM1_ESM.pdf]

# Supplementary Materials

**Title:** Superior Canal Dehiscence Syndrome Induces Canal-Specific Kinematic Adaptations During Locomotion

**Authors:** Raabeae Aryan[1], Jennifer L. Millar[2], Chenhao Bao[1], John P. Carey[3], Michael C. Schubert[2,3], Kathleen E. Cullen[1,3,4,5,*]

**Affiliations: 1)** Department of Biomedical Engineering, Johns Hopkins University School of Medicine, Baltimore, MD, United States; **2)** Department of Physical Medicine and Rehabilitation, Johns Hopkins University School of Medicine, Baltimore, MD, United States; **3)** Department of Otolaryngology–Head and Neck Surgery, Johns Hopkins University School of Medicine, Baltimore, MD, United States; **4)** Department of Neuroscience, Johns Hopkins University School of Medicine, Baltimore, MD, United States; **5)** Kavli Neuroscience Discovery Institute, Johns Hopkins University, Baltimore, MD, United States.

[*] **Corresponding Author:** Dr. Kathleen E. Cullen; Email: Kathleen.Cullen@jhu.edu

**Conflict of Interests:** None.

# Supplementary Figure 1

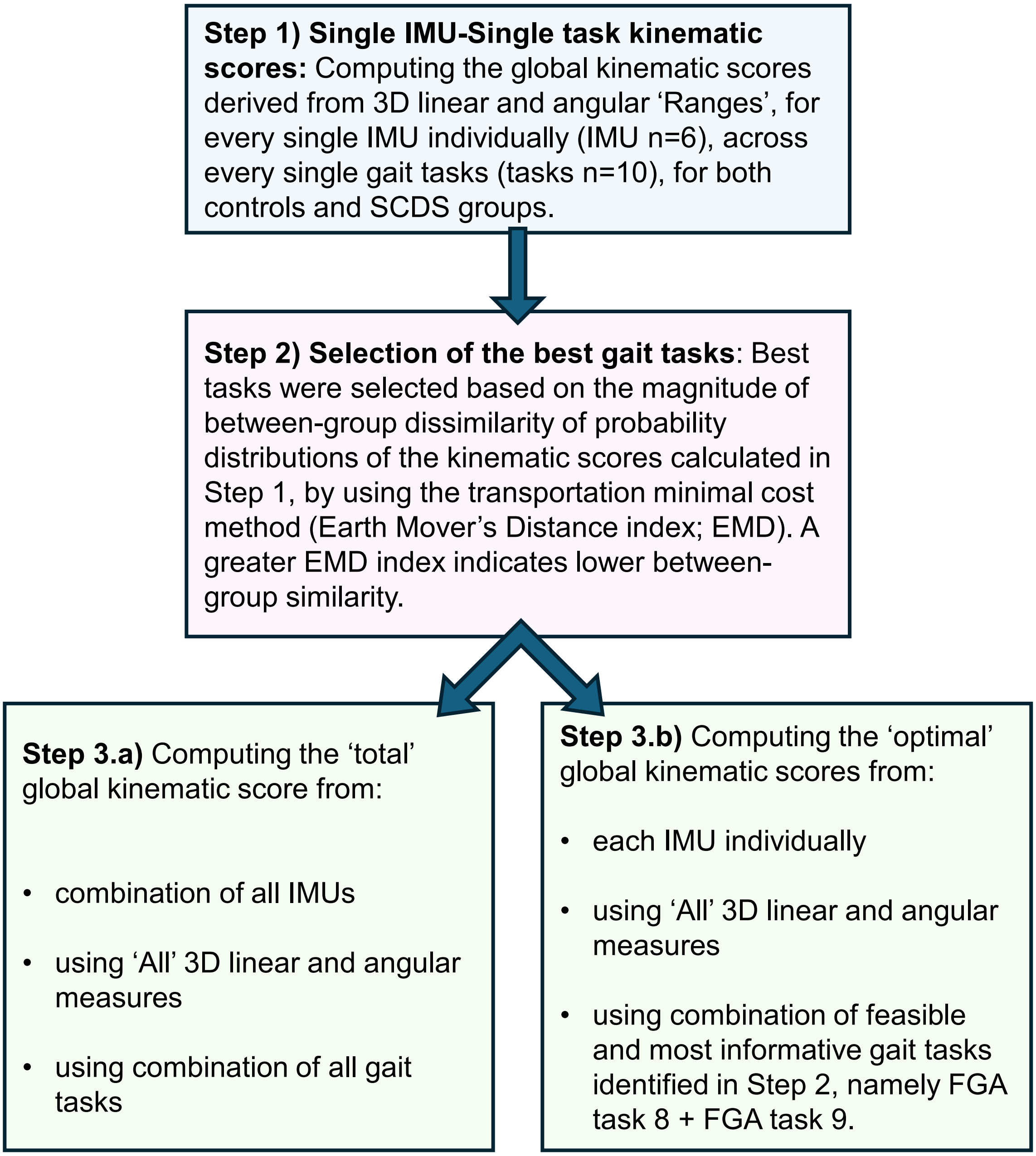


**Supplementary Figure 1:** Schematic process of optimizing selection of the most informative gait tasks, and steps for computing the global kinematic scores. FGA: Functional Gait Assessment scale. IMU: inertial measurement units. SCDS: superior canal dehiscence syndrome. Numerical values of different septs of this process, for both controls and SCDS groups, have been presented in Supplementary Tables 11-13.

# Supplementary Figure 2

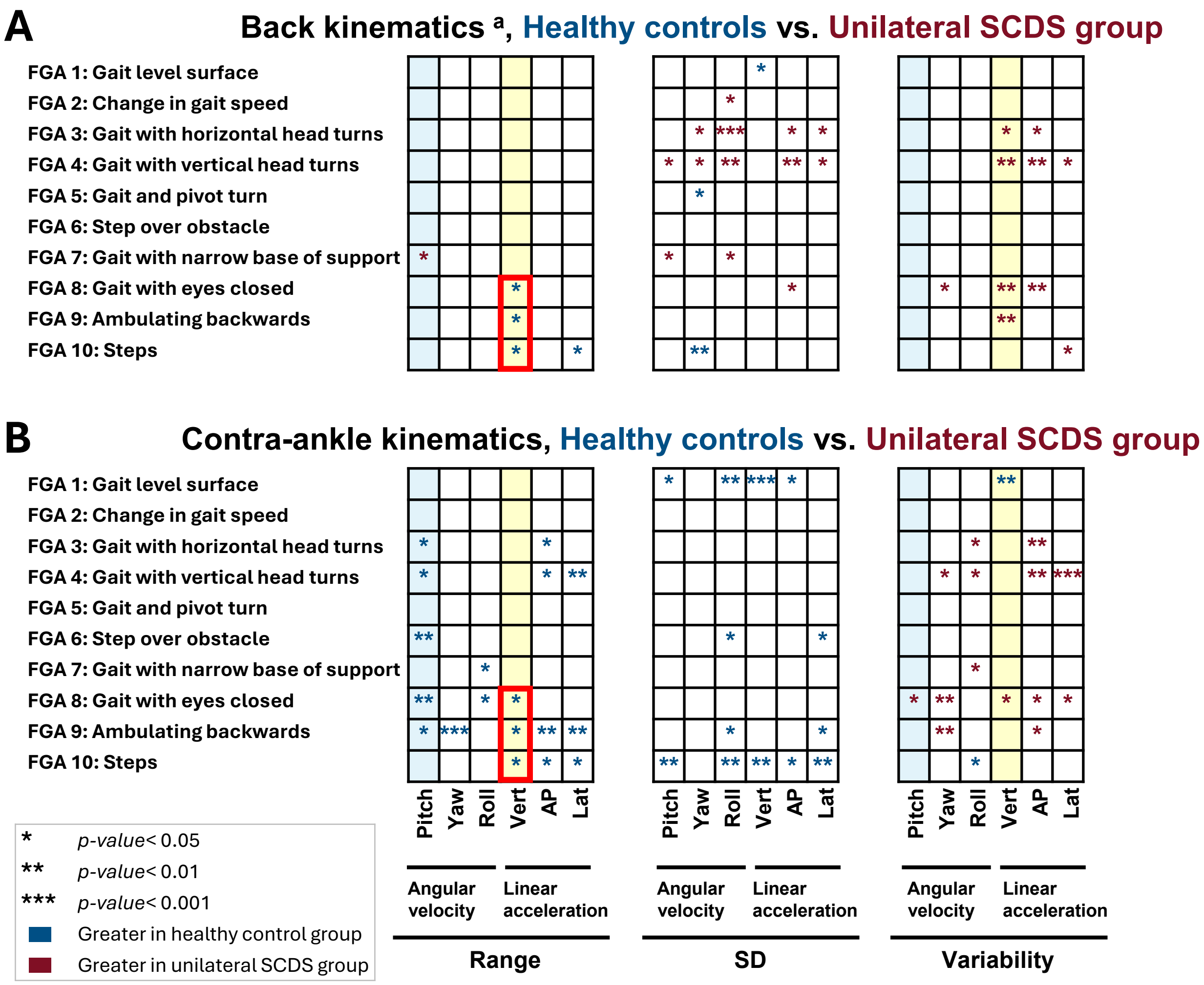


**Supplementary Figure 2:** Between-group comparisons of angular velocities (pitch, yaw, roll) and linear accelerations (vertical, anteroposterior, lateral) of (**A**) Back IMU, and (**B**) Contra-lesional Ankle IMU, during all 10 items of the Functional Gait Assessment (FGA) scale. Blue asterisks indicate statistically significant differences with greater values for the healthy control group, and red asterisks indicate statistically significant differences with greater values for the SCDS group. Light blue columns are pitch range and pitch variability; light yellow columns are vertical range and vertical variability. The red box highlights the gait tasks consistently showing a reduced range of vertical acceleration in the SCDS group. * $p < 0.05$, ** $p < 0.01$, and *** $p < 0.001$. SD: standard deviation. Numerical values (mean ± SD) of each kinematic measure for both controls and SCDS groups have been presented in Supplementary Tables 8 to 10. [a]: Back IMU data was available for 16 individuals with SCDS and 15 healthy controls.

# Supplementary Figure 3

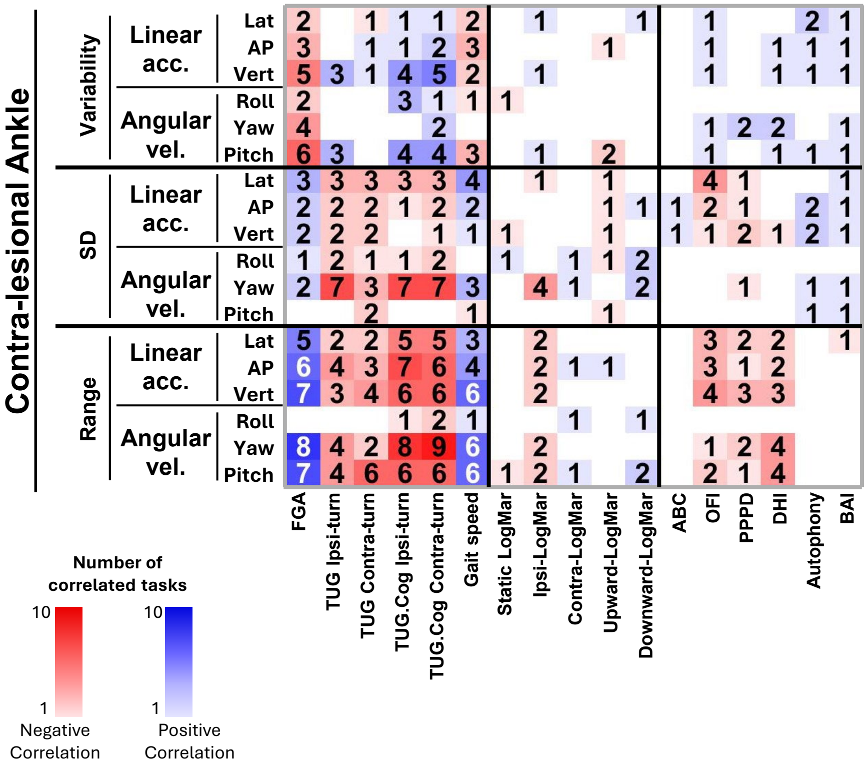


**Supplementary Figure 3:** Correlations among the clinical measures of individual with SCDS (x-axis) and their kinematic gait measures (y-axis) calculated for contra-lesional ankle IMU. Blue and red squares reflect positive and negative correlations, respectively. Brightness and the numbers in each cell of the matrix indicate the number of tasks (out of 10) in which there was a significant correlation between the kinematic and clinical measures (p-value< 0.05). SD: standard deviation. acc: acceleration. vel: velocity. Ipsi: ipsi-lesional. Contra: contra-lesional. FGA: Functional Gait Assessment scale. TUG: Timed Up and Go test. TUG.Cog: cognitive (dual task) Timed Up and Go test. Lat: lateral. Vert: vertical. AP: anteroposterior. OFI: Oscillopsia Functional Impact questionnaire. DHI: Dizziness Handicap Inventory questionnaire. BAI: Beck Anxiety Index questionnaire. PPPD: Niigata Persistent Postural-Perceptual Dizziness questionnaire. DHI: Dizziness Handicap Inventory questionnaire. Autophony: Autophony Index questionnaire. ABC: Activities-specific Balance Confidence questionnaire.

**Supplementary Table 1:** Mean ± SD of 'range' of 3D angular velocities and 3D linear accelerations during 10 gait tasks of the FGA scale, recorded from IMUs on the head and waist.

| | | Range of Angular Velocities (deg/s) | | | | | | Range of Linear Accelerations (g) | | | | | |
|---|---|---|---|---|---|---|---|---|---|---|---|---|---|
| IMU | FGA Tasks | SCDS Group | | | Control Group | | | SCDS Group | | | Control Group | | |
| | | Pitch | Yaw | Roll | Pitch | Yaw | Roll | Vert | AP | Lat | Vert | AP | Lat |
| HEAD | FGA 1 | 42 ± 9 | 34 ± 14 | 33 ± 15 | 53 ± 17* | 42 ± 17 | 40 ± 14 | 0.70 ± 0.17 | 0.28 ± 0.08 | 0.29 ± 0.13 | 0.88 ± 0.23* | 0.33 ± 0.10 | 0.32 ± 0.15 |
| | FGA 2 | 43 ± 16 | 30 ± 10 | 26 ± 9 | 47 ± 15 | 36 ± 13 | 28 ± 13 | 0.72 ± 0.28 | 0.27 ± 0.10 | 0.26 ± 0.12 | 0.81 ± 0.17 | 0.31 ± 0.11 | 0.23 ± 0.07 |
| | FGA 3 | 28 ± 9 | 141 ± 36 | 42 ± 17 | 32 ± 9 | 133 ± 30 | 47 ± 21 | 0.56 ± 0.15 | 0.21 ± 0.06 | 0.29 ± 0.09 | 0.71 ± 0.19* | 0.30 ± 0.09** | 0.27 ± 0.10 |
| | FGA 4 | 98 ± 27 | 28 ± 8 | 27 ± 13 | 95 ± 27 | 30 ± 10 | 24 ± 10 | 0.52 ± 0.16 | 0.51 ± 0.14 | 0.24 ± 0.09 | 0.66 ± 0.14* | 0.50 ± 0.12 | 0.22 ± 0.08 |
| | FGA 5 | 36 ± 9 | 43 ± 24 | 26 ± 9 | 48 ± 11** | 53 ± 19 | 31 ± 14 | 0.59 ± 0.13 | 0.27 ± 0.08 | 0.22 ± 0.10 | 0.68 ± 0.17 | 0.32 ± 0.10 | 0.24 ± 0.08 |
| | FGA 6 | 47 ± 14 | 28 ± 9 | 25 ± 10 | 58 ± 23 | 41 ± 11** | 27 ± 9 | 0.61 ± 0.18 | 0.32 ± 0.12 | 0.28 ± 0.11 | 0.77 ± 0.22* | 0.36 ± 0.11 | 0.28 ± 0.09 |
| | FGA 7 | 23 ± 9 | 21 ± 6 | 21 ± 7 | 24 ± 9 | 21 ± 6 | 17 ± 6 | 0.16 ± 0.06 | 0.18 ± 0.08 | 0.17 ± 0.09 | 0.17 ± 0.08 | 0.16 ± 0.05 | 0.13 ± 0.05 |
| | FGA 8 | 21 ± 6 | 28 ± 7 | 16 ± 5 | 30 ± 9** | 32 ± 8 | 15 ± 5 | 0.24 ± 0.11 | 0.14 ± 0.06 | 0.15 ± 0.07 | 0.39 ± 0.14** | 0.19 ± 0.06* | 0.13 ± 0.03 |
| | FGA 9 | 34 ± 15 | 27 ± 16 | 23 ± 11 | 41 ± 15 | 35 ± 14 | 26 ± 12 | 0.26 ± 0.14 | 0.15 ± 0.07 | 0.19 ± 0.09 | 0.45 ± 0.21** | 0.22 ± 0.08** | 0.22 ± 0.08 |
| | FGA 10 | 33 ± 7 | 28 ± 9 | 26 ± 13 | 50 ± 16*** | 39 ± 12** | 28 ± 7 | 0.61 ± 0.13 | 0.25 ± 0.07 | 0.25 ± 0.10 | 0.80 ± 0.24** | 0.25 ± 0.07 | 0.28 ± 0.09 |
| WAIST | FGA 1 | 122 ± 72 | 77 ± 24 | 83 ± 27 | 121 ± 97 | 74 ± 21 | 83 ± 21 | 0.83 ± 0.28 | 0.64 ± 0.21 | 0.51 ± 0.12 | 0.90 ± 0.31 | 0.79 ± 0.36 | 0.58 ± 0.28 |
| | FGA 2 | 118 ± 66 | 76 ± 25 | 75 ± 26 | 91 ± 55 | 73 ± 21 | 66 ± 12 | 0.75 ± 0.24 | 0.63 ± 0.20 | 0.55 ± 0.23 | 0.80 ± 0.22 | 0.68 ± 0.28 | 0.54 ± 0.21 |
| | FGA 3 | 107 ± 77 | 70 ± 18 | 70 ± 25 | 102 ± 74 | 72 ± 28 | 71 ± 16 | 0.64 ± 0.22 | 0.52 ± 0.17 | 0.47 ± 0.16 | 0.78 ± 0.27 | 0.70 ± 0.29* | 0.52 ± 0.18 |
| | FGA 4 | 114 ± 76 | 67 ± 22 | 73 ± 28 | 99 ± 58 | 73 ± 26 | 75 ± 15 | 0.71 ± 0.25 | 0.55 ± 0.15 | 0.47 ± 0.21 | 0.78 ± 0.21 | 0.66 ± 0.24 | 0.54 ± 0.19 |
| | FGA 5 | 118 ± 75 | 74 ± 23 | 72 ± 20 | 87 ± 46 | 78 ± 26 | 68 ± 14 | 0.70 ± 0.20 | 0.55 ± 0.16 | 0.40 ± 0.13 | 0.71 ± 0.22 | 0.61 ± 0.19 | 0.47 ± 0.15 |
| | FGA 6 | 109 ± 60 | 72 ± 30 | 59 ± 21 | 113 ± 65 | 86 ± 19 | 59 ± 15 | 0.72 ± 0.26 | 0.58 ± 0.16 | 0.45 ± 0.20 | 0.78 ± 0.20 | 0.76 ± 0.31* | 0.55 ± 0.22 |
| | FGA 7 | 45 ± 22 | 40 ± 11 | 26 ± 6 | 38 ± 19 | 43 ± 13 | 27 ± 12 | 0.18 ± 0.04 | 0.22 ± 0.06 | 0.19 ± 0.07 | 0.20 ± 0.12 | 0.20 ± 0.06 | 0.20 ± 0.10 |
| | FGA 8 | 53 ± 25 | 46 ± 16 | 41 ± 18 | 65 ± 50 | 53 ± 14 | 54 ± 13* | 0.29 ± 0.16 | 0.26 ± 0.10 | 0.23 ± 0.07 | 0.42 ± 0.17* | 0.38 ± 0.15** | 0.33 ± 0.10** |
| | FGA 9 | 49 ± 18 | 53 ± 24 | 38 ± 16 | 67 ± 28* | 66 ± 18 | 51 ± 11* | 0.28 ± 0.15 | 0.36 ± 0.13 | 0.29 ± 0.12 | 0.43 ± 0.19* | 0.45 ± 0.17 | 0.37 ± 0.11* |
| | FGA 10 | 78 ± 46 | 42 ± 12 | 38 ± 16 | 108 ± 97 | 55 ± 24* | 47 ± 17 | 0.64 ± 0.14 | 0.42 ± 0.14 | 0.36 ± 0.15 | 0.80 ± 0.26* | 0.58 ± 0.25* | 0.53 ± 0.44 |

**SD:** standard deviation. **IMU:** inertial measurement unit. **FGA:** Functional Gait Assessment scale. **Vert:** vertical acceleration. **AP:** antero-posterior acceleration. **Lat:** lateral acceleration. **FGA1:** Gait level surface. **FGA2:** Change in gait speed. **FGA3:** Gait with horizontal head turns. **FGA4:** Gait with vertical head turns. **FGA5:** Gait and pivot turn. **FGA6:** Step over obstacle. **FGA7:** Gait with narrow base of support. **FGA8:** Gait with eyes closed. **FGA9:** Ambulating backwards. **FGA10:** Steps. Blue asterisks indicate significantly greater values for the healthy control group. Red asterisks indicate significantly greater values for the SCDS group. * $p<0.05$, ** $p<0.01$, *** $p<0.001$.

**Supplementary Table 2:** Mean ± SD of 'standard deviation' of 3D angular velocities and 3D linear accelerations during 10 gait tasks of the FGA scale, recorded from IMUs on the head and waist.

| | | Standard Deviation of Angular Velocities (deg/s) | | | | | | Standard Deviation of Linear Accelerations (g) | | | | | |
|---|---|---|---|---|---|---|---|---|---|---|---|---|---|
| IMU | FGA Tasks | SCDS Group | | | Control Group | | | SCDS Group | | | Control Group | | |
| | | Pitch | Yaw | Roll | Pitch | Yaw | Roll | Vert | AP | Lat | Vert | AP | Lat |
| HEAD | FGA 1 | 12 ± 5 | 8 ± 4 | 6 ± 1 | 14 ± 5 | 8 ± 4 | 7 ± 2 | 0.08 ± 0.02 | 0.11 ± 0.06 | 0.04 ± 0.01 | 0.10 ± 0.03* | 0.14 ± 0.07 | 0.04 ± 0.01 |
| | FGA 2 | 15 ± 4 | 8 ± 2 | 8 ± 2 | 16 ± 5 | 10 ± 2** | 9 ± 2 | 0.17 ± 0.06 | 0.14 ± 0.04 | 0.06 ± 0.02 | 0.19 ± 0.04 | 0.13 ± 0.03 | 0.06 ± 0.02 |
| | FGA 3 | 14 ± 5 | 90 ± 19 | 24 ± 9 | 12 ± 3 | 94 ± 16 | 28 ± 12 | 0.08 ± 0.02 | 0.12 ± 0.04 | 0.11 ± 0.04 | 0.08 ± 0.02 | 0.11 ± 0.04 | 0.11 ± 0.03 |
| | FGA 4 | 60 ± 16 | 9 ± 3 | 12 ± 7* | 56 ± 14 | 8 ± 3 | 8 ± 2 | 0.18 ± 0.05 | 0.55 ± 0.12 | 0.08 ± 0.05** | 0.16 ± 0.04 | 0.48 ± 0.10 | 0.05 ± 0.02 |
| | FGA 5 | 11 ± 5 | 10 ± 6 | 6 ± 2 | 12 ± 3 | 17 ± 11* | 8 ± 3* | 0.08 ± 0.03 | 0.10 ± 0.04 | 0.04 ± 0.02 | 0.10 ± 0.02 | 0.11 ± 0.07 | 0.04 ± 0.02 |
| | FGA 6 | 15 ± 4 | 7 ± 2 | 7 ± 2 | 17 ± 4 | 10 ± 4* | 8 ± 2 | 0.11 ± 0.02 | 0.12 ± 0.04 | 0.06 ± 0.02 | 0.14 ± 0.04* | 0.15 ± 0.04 | 0.06 ± 0.02 |
| | FGA 7 | 8 ± 3 | 7 ± 3 | 8 ± 4* | 8 ± 3 | 6 ± 2 | 6 ± 2 | 0.05 ± 0.02 | 0.07 ± 0.04 | 0.07 ± 0.04* | 0.05 ± 0.02 | 0.08 ± 0.05 | 0.04 ± 0.02 |
| | FGA 8 | 7 ± 2 | 6 ± 1 | 5 ± 2 | 7 ± 2 | 5 ± 1 | 4 ± 1 | 0.05 ± 0.01 | 0.05 ± 0.01 | 0.03 ± 0.01* | 0.06 ± 0.02 | 0.04 ± 0.01 | 0.02 ± 0.01 |
| | FGA 9 | 9 ± 4 | 6 ± 3 | 5 ± 1 | 11 ± 4 | 7 ± 5 | 5 ± 2 | 0.05 ± 0.01 | 0.06 ± 0.02 | 0.03 ± 0.01 | 0.07 ± 0.03* | 0.07 ± 0.03* | 0.04 ± 0.01 |
| | FGA 10 | 16 ± 6 | 16 ± 6 | 10 ± 3 | 17 ± 5 | 26 ± 8*** | 12 ± 3* | 0.16 ± 0.04 | 0.16 ± 0.04 | 0.06 ± 0.02 | 0.21 ± 0.06** | 0.16 ± 0.05 | 0.06 ± 0.01 |
| WAIST | FGA 1 | 17 ± 9 | 10 ± 3 | 8 ± 1 | 18 ± 10 | 13 ± 3* | 9 ± 2* | 0.09 ± 0.03 | 0.07 ± 0.02 | 0.07 ± 0.02 | 0.11 ± 0.03 | 0.09 ± 0.03 | 0.09 ± 0.03 |
| | FGA 2 | 38 ± 25 | 21 ± 7 | 17 ± 5* | 29 ± 12 | 22 ± 5 | 14 ± 2 | 0.20 ± 0.07 | 0.16 ± 0.06 | 0.15 ± 0.06 | 0.21 ± 0.06 | 0.16 ± 0.04 | 0.15 ± 0.04 |
| | FGA 3 | 19 ± 9 | 14 ± 4 | 9 ± 2 | 14 ± 6 | 13 ± 2 | 7 ± 1 | 0.09 ± 0.02 | 0.08 ± 0.02 | 0.08 ± 0.02 | 0.09 ± 0.03 | 0.07 ± 0.02 | 0.08 ± 0.02 |
| | FGA 4 | 20 ± 12 | 11 ± 2 | 8 ± 2** | 15 ± 8 | 11 ± 2 | 7 ± 1 | 0.09 ± 0.02 | 0.08 ± 0.03 | 0.07 ± 0.01 | 0.08 ± 0.03 | 0.07 ± 0.02 | 0.07 ± 0.02 |
| | FGA 5 | 19 ± 11 | 14 ± 5 | 9 ± 3 | 16 ± 8 | 20 ± 7** | 11 ± 4 | 0.09 ± 0.03 | 0.08 ± 0.03 | 0.07 ± 0.02 | 0.11 ± 0.03 | 0.09 ± 0.03 | 0.09 ± 0.02 |
| | FGA 6 | 32 ± 14 | 17 ± 4 | 14 ± 3 | 27 ± 11 | 22 ± 6* | 15 ± 3 | 0.13 ± 0.03 | 0.14 ± 0.03 | 0.11 ± 0.03 | 0.16 ± 0.05 | 0.15 ± 0.04 | 0.13 ± 0.03 |
| | FGA 7 | 12 ± 6 | 11 ± 4 | 8 ± 2 | 9 ± 5 | 9 ± 3 | 6 ± 2 | 0.06 ± 0.02 | 0.06 ± 0.03 | 0.06 ± 0.02 | 0.06 ± 0.03 | 0.05 ± 0.02 | 0.05 ± 0.02 |
| | FGA 8 | 13 ± 5 | 10 ± 2 | 7 ± 2 | 12 ± 6 | 9 ± 2 | 7 ± 2 | 0.06 ± 0.02 | 0.06 ± 0.02 | 0.05 ± 0.01 | 0.07 ± 0.03 | 0.06 ± 0.01 | 0.06 ± 0.02 |
| | FGA 9 | 12 ± 4 | 9 ± 2 | 7 ± 2 | 12 ± 6 | 11 ± 3* | 7 ± 2 | 0.06 ± 0.02 | 0.06 ± 0.01 | 0.05 ± 0.01 | 0.07 ± 0.03 | 0.06 ± 0.03 | 0.06 ± 0.02 |
| | FGA 10 | 30 ± 15 | 23 ± 7 | 18 ± 6 | 28 ± 16 | 30 ± 7** | 20 ± 5 | 0.19 ± 0.06 | 0.15 ± 0.04 | 0.13 ± 0.04 | 0.24 ± 0.06* | 0.16 ± 0.04 | 0.16 ± 0.04* |

**SD:** standard deviation. **IMU:** inertial measurement unit. **FGA:** Functional Gait Assessment scale. **Vert:** vertical acceleration. **AP:** antero-posterior acceleration. **Lat:** lateral acceleration. **FGA1:** Gait level surface. **FGA2:** Change in gait speed. **FGA3:** Gait with horizontal head turns. **FGA4:** Gait with vertical head turns. **FGA5:** Gait and pivot turn. **FGA6:** Step over obstacle. **FGA7:** Gait with narrow base of support. **FGA8:** Gait with eyes closed. **FGA9:** Ambulating backwards. **FGA10:** Steps. Blue asterisks indicate significantly greater values for the healthy control group. Red asterisks indicate significantly greater values for the SCDS group. * $p<0.05$, ** $p<0.01$, *** $p<0.001$.

**Supplementary Table 3:** Mean ± SD of 'variability' of 3D angular velocities and 3D linear accelerations during 10 gait tasks of the FGA scale, recorded from IMUs on the head and waist.

| | | Variability of Angular Velocities | | | | | | Variability of Linear Accelerations | | | | | |
|---|---|---|---|---|---|---|---|---|---|---|---|---|---|
| IMU | FGA Tasks | SCDS Group | | | Control Group | | | SCDS Group | | | Control Group | | |
| | | Pitch | Yaw | Roll | Pitch | Yaw | Roll | Vert | AP | Lat | Vert | AP | Lat |
| HEAD | FGA 1 | 0.28 ± 0.11 | 0.25 ± 0.14 | 0.19 ± 0.08 | 0.27 ± 0.09 | 0.21 ± 0.07 | 0.18 ± 0.05 | 0.12 ± 0.03 | 0.42 ± 0.24 | 0.15 ± 0.07 | 0.11 ± 0.02 | 0.44 ± 0.26 | 0.14 ± 0.05 |
| | FGA 2 | 0.36 ± 0.09 | 0.28 ± 0.07 | 0.34 ± 0.08 | 0.37 ± 0.13 | 0.29 ± 0.07 | 0.35 ± 0.11 | 0.25 ± 0.05 | 0.58 ± 0.24 | 0.25 ± 0.06 | 0.24 ± 0.04 | 0.47 ± 0.21 | 0.26 ± 0.05 |
| | FGA 3 | 0.50 ± 0.11*** | 0.67 ± 0.21 | 0.59 ± 0.15 | 0.38 ± 0.06 | 0.73 ± 0.17 | 0.62 ± 0.12 | 0.16 ± 0.07 | 0.59 ± 0.22* | 0.40 ± 0.12 | 0.12 ± 0.03 | 0.40 ± 0.18 | 0.41 ± 0.08 |
| | FGA 4 | 0.64 ± 0.17 | 0.34 ± 0.13 | 0.48 ± 0.14 | 0.62 ± 0.17 | 0.29 ± 0.09 | 0.38 ± 0.13 | 0.39 ± 0.18** | 1.14 ± 0.37 | 0.36 ± 0.17* | 0.25 ± 0.09 | 1.02 ± 0.34 | 0.23 ± 0.10 |
| | FGA 5 | 0.31 ± 0.12 | 0.25 ± 0.14 | 0.23 ± 0.09 | 0.26 ± 0.08 | 0.31 ± 0.13 | 0.26 ± 0.07 | 0.14 ± 0.04 | 0.38 ± 0.11 | 0.18 ± 0.08 | 0.15 ± 0.04 | 0.39 ± 0.30 | 0.19 ± 0.06 |
| | FGA 6 | 0.33 ± 0.10 | 0.27 ± 0.07 | 0.34 ± 0.14 | 0.31 ± 0.09 | 0.24 ± 0.06 | 0.32 ± 0.09 | 0.19 ± 0.05 | 0.43 ± 0.21 | 0.22 ± 0.08 | 0.18 ± 0.04 | 0.45 ± 0.17 | 0.21 ± 0.06 |
| | FGA 7 | 0.34 ± 0.11 | 0.35 ± 0.09 | 0.41 ± 0.11 | 0.33 ± 0.08 | 0.30 ± 0.08 | 0.36 ± 0.09 | 0.34 ± 0.08 | 0.40 ± 0.12 | 0.41 ± 0.07 | 0.30 ± 0.07 | 0.53 ± 0.26 | 0.35 ± 0.11 |
| | FGA 8 | 0.35 ± 0.09** | 0.23 ± 0.09* | 0.31 ± 0.11 | 0.25 ± 0.09 | 0.17 ± 0.04 | 0.27 ± 0.08 | 0.26 ± 0.08** | 0.38 ± 0.18** | 0.25 ± 0.12 | 0.18 ± 0.05 | 0.24 ± 0.07 | 0.19 ± 0.05 |
| | FGA 9 | 0.30 ± 0.12 | 0.23 ± 0.07 | 0.26 ± 0.13 | 0.30 ± 0.17 | 0.21 ± 0.08 | 0.24 ± 0.13 | 0.24 ± 0.08** | 0.43 ± 0.18 | 0.21 ± 0.08 | 0.18 ± 0.04 | 0.37 ± 0.17 | 0.17 ± 0.06 |
| | FGA 10 | 0.50 ± 0.18* | 0.60 ± 0.25 | 0.45 ± 0.16 | 0.36 ± 0.10 | 0.72 ± 0.30 | 0.46 ± 0.12 | 0.27 ± 0.09 | 0.66 ± 0.22 | 0.26 ± 0.08 | 0.28 ± 0.08 | 0.64 ± 0.20 | 0.22 ± 0.09 |
| WAIST | FGA 1 | 0.17 ± 0.07 | 0.15 ± 0.06 | 0.10 ± 0.04 | 0.17 ± 0.05 | 0.18 ± 0.04 | 0.11 ± 0.02 | 0.11 ± 0.04 | 0.12 ± 0.03 | 0.14 ± 0.05 | 0.12 ± 0.02 | 0.12 ± 0.02 | 0.16 ± 0.04 |
| | FGA 2 | 0.32 ± 0.09 | 0.28 ± 0.05 | 0.24 ± 0.06 | 0.34 ± 0.08 | 0.31 ± 0.07 | 0.21 ± 0.03 | 0.27 ± 0.06 | 0.26 ± 0.06 | 0.29 ± 0.09 | 0.27 ± 0.07 | 0.24 ± 0.06 | 0.30 ± 0.09 |
| | FGA 3 | 0.22 ± 0.11 | 0.22 ± 0.07 | 0.15 ± 0.08 | 0.17 ± 0.04 | 0.19 ± 0.05 | 0.11 ± 0.03 | 0.15 ± 0.06* | 0.16 ± 0.06*** | 0.18 ± 0.07 | 0.12 ± 0.03 | 0.11 ± 0.02 | 0.16 ± 0.05 |
| | FGA 4 | 0.20 ± 0.07 | 0.18 ± 0.07 | 0.13 ± 0.06* | 0.16 ± 0.05 | 0.16 ± 0.05 | 0.09 ± 0.02 | 0.14 ± 0.04* | 0.15 ± 0.05*** | 0.18 ± 0.07 | 0.11 ± 0.02 | 0.11 ± 0.02 | 0.14 ± 0.04 |
| | FGA 5 | 0.18 ± 0.06 | 0.19 ± 0.07 | 0.13 ± 0.04 | 0.20 ± 0.05 | 0.27 ± 0.11* | 0.16 ± 0.06 | 0.14 ± 0.05 | 0.15 ± 0.05 | 0.19 ± 0.06 | 0.16 ± 0.05 | 0.15 ± 0.03 | 0.19 ± 0.04 |
| | FGA 6 | 0.34 ± 0.13 | 0.26 ± 0.08 | 0.26 ± 0.07 | 0.27 ± 0.08 | 0.25 ± 0.05 | 0.27 ± 0.07 | 0.19 ± 0.04 | 0.25 ± 0.06* | 0.27 ± 0.09 | 0.21 ± 0.04 | 0.20 ± 0.05 | 0.25 ± 0.07 |
| | FGA 7 | 0.28 ± 0.09 | 0.27 ± 0.08 | 0.30 ± 0.08 | 0.25 ± 0.07 | 0.23 ± 0.07 | 0.26 ± 0.10 | 0.33 ± 0.08 | 0.29 ± 0.08 | 0.31 ± 0.08 | 0.31 ± 0.11 | 0.26 ± 0.13 | 0.27 ± 0.07 |
| | FGA 8 | 0.28 ± 0.10* | 0.24 ± 0.09 | 0.21 ± 0.09** | 0.21 ± 0.06 | 0.19 ± 0.06 | 0.13 ± 0.05 | 0.23 ± 0.08** | 0.27 ± 0.16** | 0.25 ± 0.08 | 0.17 ± 0.04 | 0.16 ± 0.04 | 0.19 ± 0.09 |
| | FGA 9 | 0.26 ± 0.06*** | 0.19 ± 0.08 | 0.19 ± 0.05** | 0.18 ± 0.05 | 0.16 ± 0.04 | 0.14 ± 0.03 | 0.24 ± 0.08* | 0.17 ± 0.06 | 0.20 ± 0.05 | 0.18 ± 0.06 | 0.14 ± 0.03 | 0.17 ± 0.06 |
| | FGA 10 | 0.42 ± 0.12** | 0.56 ± 0.14 | 0.50 ± 0.16 | 0.31 ± 0.10 | 0.61 ± 0.21 | 0.45 ± 0.14 | 0.30 ± 0.07 | 0.38 ± 0.14** | 0.39 ± 0.10 | 0.31 ± 0.09 | 0.29 ± 0.06 | 0.36 ± 0.09 |

**SD:** standard deviation. **IMU:** inertial measurement unit. **FGA:** Functional Gait Assessment scale. **Vert:** vertical acceleration. **AP:** antero-posterior acceleration. **Lat:** lateral acceleration. **FGA1:** Gait level surface. **FGA2:** Change in gait speed. **FGA3:** Gait with horizontal head turns. **FGA4:** Gait with vertical head turns. **FGA5:** Gait and pivot turn. **FGA6:** Step over obstacle. **FGA7:** Gait with narrow base of support. **FGA8:** Gait with eyes closed. **FGA9:** Ambulating backwards. **FGA10:** Steps. Blue asterisks indicate significantly greater values for the healthy control group. Red asterisks indicate significantly greater values for the SCDS group. * p<0.05, ** p<0.01, *** p<0.001.

**Supplementary Table 4:** Mean ± SD of 'range' of 3D angular velocities and 3D linear accelerations during 10 gait tasks of the FGA scale, recorded from IMUs on the ipsi-lesional ankle and dominant wrist.

| | | **Range of Angular Velocities (deg/s)** | | | | | | **Range of Linear Accelerations (g)** | | | | | |
|---|---|---|---|---|---|---|---|---|---|---|---|---|---|
| **IMU** | **FGA Tasks** | **SCDS Group** | | | **Control Group** | | | **SCDS Group** | | | **Control Group** | | |
| | | **Pitch** | **Yaw** | **Roll** | **Pitch** | **Yaw** | **Roll** | **Vert** | **AP** | **Lat** | **Vert** | **AP** | **Lat** |
| **IPSI-LESIONAL ANKLE** | **FGA 1** | 578 ± 55 | 213 ± 46 | 127 ± 47 | 619 ± 62 | 245 ± 69 | 167 ± 89 | 2.14 ± 0.48 | 4.83 ± 0.95 | 1.30 ± 0.48 | 2.35 ± 0.40 | 5.44 ± 1.41 | 1.76 ± 0.75* |
| | **FGA 2** | 507 ± 88 | 184 ± 43 | 115 ± 41 | 537 ± 71 | 188 ± 41 | 137 ± 69 | 1.77 ± 0.39 | 4.47 ± 0.87 | 1.10 ± 0.42 | 1.89 ± 0.47 | 4.69 ± 1.00 | 1.31 ± 0.57 |
| | **FGA 3** | 505 ± 84 | 180 ± 57 | 112 ± 45 | 575 ± 85* | 226 ± 57* | 146 ± 72 | 1.64 ± 0.51 | 4.41 ± 1.14 | 1.04 ± 0.43 | 2.08 ± 0.52* | 5.15 ± 1.54 | 1.46 ± 0.69* |
| | **FGA 4** | 517 ± 81 | 197 ± 66 | 117 ± 41 | 593 ± 72** | 226 ± 53 | 148 ± 68 | 1.73 ± 0.51 | 4.51 ± 1.22 | 1.10 ± 0.59 | 2.23 ± 0.51** | 5.14 ± 1.50 | 1.42 ± 0.63 |
| | **FGA 5** | 541 ± 77 | 210 ± 45 | 117 ± 38 | 565 ± 61 | 232 ± 57 | 140 ± 68 | 1.91 ± 0.49 | 4.31 ± 0.92 | 1.09 ± 0.40 | 2.05 ± 0.36 | 4.58 ± 1.02 | 1.33 ± 0.53 |
| | **FGA 6** | 490 ± 113 | 178 ± 59 | 108 ± 36 | 554 ± 111 | 216 ± 65 | 133 ± 63 | 1.84 ± 0.66 | 4.36 ± 1.26 | 1.02 ± 0.40 | 2.06 ± 0.54 | 4.71 ± 1.45 | 1.31 ± 0.50 |
| | **FGA 7** | 329 ± 57 | 163 ± 61 | 95 ± 37 | 372 ± 82 | 185 ± 53 | 108 ± 33 | 1.07 ± 0.35 | 2.95 ± 1.63 | 0.75 ± 0.25 | 1.30 ± 0.49 | 3.01 ± 1.08 | 0.86 ± 0.29 |
| | **FGA 8** | 377 ± 87 | 127 ± 64 | 85 ± 42 | 475 ± 71** | 181 ± 63* | 102 ± 39 | 1.23 ± 0.49 | 2.44 ± 0.86 | 0.64 ± 0.36 | 1.64 ± 0.38* | 3.21 ± 0.94* | 0.89 ± 0.44 |
| | **FGA 9** | 338 ± 70 | 127 ± 63 | 76 ± 24 | 407 ± 56** | 185 ± 51** | 108 ± 40** | 0.95 ± 0.37 | 1.86 ± 0.65 | 0.67 ± 0.26 | 1.40 ± 0.51** | 2.76 ± 0.74** | 0.99 ± 0.29** |
| | **FGA 10** | 299 ± 48 | 123 ± 33 | 72 ± 28 | 341 ± 63* | 169 ± 51** | 92 ± 34 | 1.23 ± 0.28 | 2.73 ± 1.26 | 0.92 ± 0.50 | 1.80 ± 0.70** | 4.25 ± 1.70** | 1.40 ± 0.54* |
| **WRIST** | **FGA 1** | 203 ± 74 | 186 ± 78 | 134 ± 80 | 251 ± 79 | 171 ± 67 | 155 ± 82 | 0.74 ± 0.14 | 0.35 ± 0.17 | 0.40 ± 0.11 | 0.77 ± 0.17 | 0.44 ± 0.12 | 0.37 ± 0.07 |
| | **FGA 2** | 252 ± 98 | 154 ± 67 | 115 ± 71 | 231 ± 77 | 134 ± 40 | 95 ± 51 | 0.66 ± 0.23 | 0.51 ± 0.31 | 0.42 ± 0.23* | 0.62 ± 0.14 | 0.53 ± 0.18 | 0.31 ± 0.07 |
| | **FGA 3** | 152 ± 61 | 133 ± 47 | 98 ± 50 | 187 ± 84 | 152 ± 57 | 107 ± 52 | 0.63 ± 0.18 | 0.30 ± 0.06 | 0.33 ± 0.08 | 0.67 ± 0.17 | 0.38 ± 0.16 | 0.34 ± 0.09 |
| | **FGA 4** | 140 ± 49 | 124 ± 47 | 90 ± 49 | 193 ± 92 | 143 ± 60 | 112 ± 49 | 0.63 ± 0.21 | 0.29 ± 0.08 | 0.32 ± 0.08 | 0.71 ± 0.16 | 0.35 ± 0.11 | 0.31 ± 0.05 |
| | **FGA 5** | 171 ± 54 | 148 ± 55 | 106 ± 48 | 175 ± 50 | 147 ± 49 | 106 ± 51 | 0.62 ± 0.14 | 0.31 ± 0.11 | 0.34 ± 0.07 | 0.64 ± 0.17 | 0.39 ± 0.14 | 0.30 ± 0.04 |
| | **FGA 6** | 152 ± 52 | 114 ± 43 | 87 ± 40 | 181 ± 83 | 139 ± 43 | 95 ± 42 | 0.68 ± 0.24 | 0.30 ± 0.08 | 0.32 ± 0.11 | 0.71 ± 0.20 | 0.40 ± 0.22 | 0.35 ± 0.10 |
| | **FGA 7** | 37 ± 39 | 40 ± 47 | 37 ± 30 | 28 ± 20 | 35 ± 19 | 32 ± 17 | 0.19 ± 0.12 | 0.24 ± 0.21 | 0.18 ± 0.08 | 0.18 ± 0.08 | 0.21 ± 0.16 | 0.20 ± 0.10 |
| | **FGA 8** | 76 ± 56 | 76 ± 25 | 53 ± 47 | 96 ± 52 | 109 ± 32** | 54 ± 31 | 0.32 ± 0.20 | 0.21 ± 0.07 | 0.24 ± 0.06 | 0.45 ± 0.16 | 0.22 ± 0.08 | 0.26 ± 0.05 |
| | **FGA 9** | 98 ± 32 | 97 ± 32 | 54 ± 16 | 140 ± 79 | 129 ± 32** | 70 ± 30 | 0.28 ± 0.12 | 0.23 ± 0.07 | 0.30 ± 0.09 | 0.42 ± 0.16** | 0.29 ± 0.12 | 0.34 ± 0.06 |
| | **FGA 10** | 105 ± 59 | 99 ± 42 | 75 ± 32 | 152 ± 67* | 111 ± 53 | 87 ± 46 | 0.61 ± 0.18 | 0.30 ± 0.13 | 0.34 ± 0.15 | 0.78 ± 0.23* | 0.33 ± 0.10 | 0.32 ± 0.08 |

**SD:** standard deviation. **IMU:** inertial measurement unit. **FGA:** Functional Gait Assessment scale. **Vert:** vertical acceleration. **AP:** antero-posterior acceleration. **Lat:** lateral acceleration. **FGA1:** Gait level surface. **FGA2:** Change in gait speed. **FGA3:** Gait with horizontal head turns. **FGA4:** Gait with vertical head turns. **FGA5:** Gait and pivot turn. **FGA6:** Step over obstacle. **FGA7:** Gait with narrow base of support. **FGA8:** Gait with eyes closed. **FGA9:** Ambulating backwards. **FGA10:** Steps. Blue asterisks indicate significantly greater values for the healthy control group. Red asterisks indicate significantly greater values for the SCDS group. * $p<0.05$, ** $p<0.01$, *** $p<0.001$.

**Supplementary Table 5:** Mean ± SD of 'standard deviation' of 3D angular velocities and 3D linear accelerations during 10 gait tasks of the FGA scale, recorded from IMUs on the ipsi-lesional ankle and dominant wrist.

| | | Standard Deviation of Angular Velocities (deg/s) | | | | | | Standard Deviation of Linear Accelerations (g) | | | | | |
|---|---|---|---|---|---|---|---|---|---|---|---|---|---|
| IMU | FGA Tasks | SCDS Group | | | Control Group | | | SCDS Group | | | Control Group | | |
| | | Pitch | Yaw | Roll | Pitch | Yaw | Roll | Vert | AP | Lat | Vert | AP | Lat |
| IPSI-LESIONAL ANKLE | FGA 1 | 29 ± 11 | 23 ± 6 | 11 ± 3 | 29 ± 7 | 26 ± 6 | 13 ± 4* | 0.16 ± 0.06 | 0.23 ± 0.07 | 0.11 ± 0.04 | 0.17 ± 0.04 | 0.26 ± 0.07 | 0.13 ± 0.03 |
| | FGA 2 | 77 ± 18 | 39 ± 9 | 20 ± 6 | 75 ± 16 | 37 ± 8 | 21 ± 9 | 0.36 ± 0.09 | 0.51 ± 0.14 | 0.20 ± 0.05 | 0.37 ± 0.09 | 0.53 ± 0.12 | 0.21 ± 0.06 |
| | FGA 3 | 29 ± 11 | 29 ± 5 | 14 ± 4 | 24 ± 5 | 28 ± 4 | 13 ± 3 | 0.16 ± 0.06 | 0.22 ± 0.05 | 0.14 ± 0.03 | 0.14 ± 0.03 | 0.22 ± 0.05 | 0.13 ± 0.03 |
| | FGA 4 | 26 ± 8 | 26 ± 4 | 12 ± 2 | 22 ± 6 | 25 ± 4 | 13 ± 4 | 0.14 ± 0.04 | 0.21 ± 0.03 | 0.12 ± 0.03 | 0.13 ± 0.03 | 0.22 ± 0.06 | 0.13 ± 0.03 |
| | FGA 5 | 35 ± 21 | 29 ± 8 | 13 ± 5 | 37 ± 15 | 39 ± 10** | 18 ± 5** | 0.17 ± 0.08 | 0.23 ± 0.07 | 0.12 ± 0.03 | 0.19 ± 0.06 | 0.27 ± 0.07 | 0.17 ± 0.04** |
| | FGA 6 | 62 ± 16 | 34 ± 5 | 19 ± 5 | 65 ± 15 | 41 ± 10* | 21 ± 8 | 0.29 ± 0.07 | 0.37 ± 0.07 | 0.16 ± 0.04 | 0.33 ± 0.08 | 0.45 ± 0.10* | 0.19 ± 0.05* |
| | FGA 7 | 27 ± 10 | 27 ± 6 | 11 ± 3 | 25 ± 9 | 28 ± 9 | 11 ± 4 | 0.12 ± 0.04 | 0.19 ± 0.05 | 0.12 ± 0.04 | 0.12 ± 0.06 | 0.21 ± 0.09 | 0.12 ± 0.04 |
| | FGA 8 | 27 ± 9 | 22 ± 5 | 11 ± 3 | 26 ± 10 | 24 ± 6 | 12 ± 4 | 0.12 ± 0.03 | 0.17 ± 0.04 | 0.10 ± 0.03 | 0.13 ± 0.04 | 0.19 ± 0.06 | 0.11 ± 0.03 |
| | FGA 9 | 20 ± 4 | 20 ± 4 | 9 ± 2 | 24 ± 8 | 25 ± 9* | 10 ± 4 | 0.09 ± 0.02 | 0.17 ± 0.03 | 0.09 ± 0.02 | 0.12 ± 0.04* | 0.19 ± 0.06 | 0.12 ± 0.03** |
| | FGA 10 | 67 ± 9 | 39 ± 7 | 22 ± 7 | 86 ± 13*** | 47 ± 10* | 30 ± 9** | 0.39 ± 0.10 | 0.44 ± 0.10 | 0.20 ± 0.06 | 0.51 ± 0.10** | 0.55 ± 0.07** | 0.25 ± 0.05* |
| WRIST | FGA 1 | 23 ± 10 | 26 ± 7 | 17 ± 8 | 33 ± 12* | 28 ± 8 | 21 ± 9 | 0.08 ± 0.02 | 0.06 ± 0.02 | 0.05 ± 0.01 | 0.11 ± 0.05* | 0.07 ± 0.02 | 0.05 ± 0.01 |
| | FGA 2 | 65 ± 22 | 47 ± 18 | 37 ± 17 | 78 ± 26 | 42 ± 11 | 37 ± 15 | 0.23 ± 0.13 | 0.22 ± 0.12 | 0.14 ± 0.07 | 0.22 ± 0.07 | 0.20 ± 0.09 | 0.11 ± 0.04 |
| | FGA 3 | 28 ± 8 | 31 ± 11 | 20 ± 10 | 26 ± 13 | 26 ± 11 | 17 ± 11 | 0.10 ± 0.04 | 0.09 ± 0.05 | 0.08 ± 0.06 | 0.11 ± 0.10 | 0.07 ± 0.05 | 0.06 ± 0.03 |
| | FGA 4 | 24 ± 8 | 25 ± 8* | 16 ± 7 | 23 ± 8 | 20 ± 6 | 14 ± 6 | 0.09 ± 0.03 | 0.08 ± 0.03* | 0.07 ± 0.02** | 0.08 ± 0.02 | 0.06 ± 0.02 | 0.05 ± 0.01 |
| | FGA 5 | 27 ± 8 | 29 ± 10 | 18 ± 8 | 32 ± 15 | 33 ± 11 | 21 ± 10 | 0.09 ± 0.03 | 0.07 ± 0.03 | 0.06 ± 0.02 | 0.11 ± 0.03 | 0.10 ± 0.04* | 0.07 ± 0.03 |
| | FGA 6 | 37 ± 11 | 37 ± 10 | 26 ± 11 | 42 ± 17 | 34 ± 11 | 24 ± 9 | 0.14 ± 0.03 | 0.12 ± 0.04 | 0.10 ± 0.03 | 0.15 ± 0.06 | 0.15 ± 0.09 | 0.10 ± 0.05 |
| | FGA 7 | 12 ± 8 | 12 ± 8 | 12 ± 8 | 8 ± 5 | 9 ± 4 | 9 ± 4 | 0.08 ± 0.05 | 0.09 ± 0.05 | 0.06 ± 0.03 | 0.07 ± 0.06 | 0.06 ± 0.02 | 0.06 ± 0.03 |
| | FGA 8 | 15 ± 5 | 19 ± 7 | 11 ± 5 | 17 ± 7 | 17 ± 4 | 11 ± 6 | 0.10 ± 0.10 | 0.07 ± 0.03 | 0.07 ± 0.07 | 0.09 ± 0.06 | 0.06 ± 0.04 | 0.05 ± 0.04 |
| | FGA 9 | 18 ± 5 | 23 ± 5 | 13 ± 3 | 24 ± 13 | 24 ± 8 | 13 ± 5 | 0.06 ± 0.01 | 0.06 ± 0.02 | 0.05 ± 0.01 | 0.09 ± 0.06* | 0.07 ± 0.04 | 0.05 ± 0.02 |
| | FGA 10 | 42 ± 17 | 48 ± 14 | 31 ± 15 | 44 ± 10 | 54 ± 10 | 31 ± 12 | 0.22 ± 0.11 | 0.17 ± 0.10 | 0.16 ± 0.12 | 0.22 ± 0.07 | 0.14 ± 0.04 | 0.11 ± 0.05 |

**SD:** standard deviation. **IMU:** inertial measurement unit. **FGA:** Functional Gait Assessment scale. **Vert:** vertical acceleration. **AP:** antero-posterior acceleration. **Lat:** lateral acceleration. **FGA1:** Gait level surface. **FGA2:** Change in gait speed. **FGA3:** Gait with horizontal head turns. **FGA4:** Gait with vertical head turns. **FGA5:** Gait and pivot turn. **FGA6:** Step over obstacle. **FGA7:** Gait with narrow base of support. **FGA8:** Gait with eyes closed. **FGA9:** Ambulating backwards. **FGA10:** Steps. Blue asterisks indicate significantly greater values for the healthy control group. Red asterisks indicate significantly greater values for the SCDS group. * p<0.05, ** p<0.01, *** p<0.001.

**Supplementary Table 6:** Mean ± SD of 'variability' of 3D angular velocities and 3D linear accelerations during 10 gait tasks of the FGA scale, recorded from IMUs on the ipsi-lesional ankle and dominant wrist.

| | | Variability of Angular Velocities | | | | | | Variability of Linear Accelerations | | | | | |
|---|---|---|---|---|---|---|---|---|---|---|---|---|---|
| | | SCDS Group | | | Control Group | | | SCDS Group | | | Control Group | | |
| IMU | FGA Tasks | Pitch | Yaw | Roll | Pitch | Yaw | Roll | Vert | AP | Lat | Vert | AP | Lat |
| IPSI-LESIONAL ANKLE | FGA 1 | 0.05 ± 0.02 | 0.11 ± 0.04 | 0.09 ± 0.03 | 0.05 ± 0.02 | 0.11 ± 0.03 | 0.09 ± 0.03 | 0.08 ± 0.03 | 0.05 ± 0.02 | 0.09 ± 0.03 | 0.08 ± 0.02 | 0.05 ± 0.01 | 0.08 ± 0.03 |
| | FGA 2 | 0.16 ± 0.06 | 0.22 ± 0.04 | 0.19 ± 0.06 | 0.14 ± 0.04 | 0.20 ± 0.04 | 0.17 ± 0.05 | 0.21 ± 0.06 | 0.12 ± 0.02 | 0.20 ± 0.08 | 0.21 ± 0.07 | 0.11 ± 0.02 | 0.18 ± 0.04 |
| | FGA 3 | 0.06 ± 0.03$^{*}$ | 0.18 ± 0.07$^{**}$ | 0.15 ± 0.07$^{*}$ | 0.04 ± 0.01 | 0.13 ± 0.03 | 0.10 ± 0.04 | 0.11 ± 0.05$^{*}$ | 0.05 ± 0.02 | 0.15 ± 0.06$^{*}$ | 0.07 ± 0.02 | 0.05 ± 0.01 | 0.10 ± 0.04 |
| | FGA 4 | 0.05 ± 0.03$^{*}$ | 0.15 ± 0.05 | 0.11 ± 0.04 | 0.04 ± 0.01 | 0.12 ± 0.03 | 0.09 ± 0.03 | 0.09 ± 0.04$^{**}$ | 0.05 ± 0.02 | 0.13 ± 0.05$^{*}$ | 0.06 ± 0.01 | 0.04 ± 0.01 | 0.10 ± 0.03 |
| | FGA 5 | 0.07 ± 0.05 | 0.15 ± 0.06 | 0.12 ± 0.05 | 0.07 ± 0.03 | 0.17 ± 0.04 | 0.15 ± 0.07 | 0.09 ± 0.05 | 0.06 ± 0.02 | 0.13 ± 0.07 | 0.09 ± 0.03 | 0.06 ± 0.01 | 0.14 ± 0.05 |
| | FGA 6 | 0.14 ± 0.05 | 0.21 ± 0.06 | 0.19 ± 0.07 | 0.12 ± 0.04 | 0.20 ± 0.04 | 0.18 ± 0.07 | 0.17 ± 0.06 | 0.09 ± 0.02 | 0.17 ± 0.04 | 0.17 ± 0.05 | 0.10 ± 0.02 | 0.15 ± 0.03 |
| | FGA 7 | 0.08 ± 0.04 | 0.19 ± 0.07 | 0.14 ± 0.07$^{*}$ | 0.07 ± 0.03 | 0.16 ± 0.04 | 0.10 ± 0.03 | 0.12 ± 0.04 | 0.08 ± 0.04 | 0.17 ± 0.06 | 0.10 ± 0.03 | 0.08 ± 0.04 | 0.14 ± 0.04 |
| | FGA 8 | 0.08 ± 0.04$^{*}$ | 0.21 ± 0.09$^{**}$ | 0.16 ± 0.11 | 0.06 ± 0.02 | 0.14 ± 0.03 | 0.12 ± 0.04 | 0.12 ± 0.06$^{*}$ | 0.08 ± 0.03$^{*}$ | 0.18 ± 0.08 | 0.08 ± 0.02 | 0.06 ± 0.02 | 0.14 ± 0.05 |
| | FGA 9 | 0.06 ± 0.02 | 0.18 ± 0.07$^{*}$ | 0.12 ± 0.04 | 0.06 ± 0.02 | 0.14 ± 0.04 | 0.10 ± 0.03 | 0.10 ± 0.02 | 0.10 ± 0.03$^{**}$ | 0.15 ± 0.05 | 0.09 ± 0.04 | 0.07 ± 0.02 | 0.12 ± 0.02 |
| | FGA 10 | 0.23 ± 0.04 | 0.32 ± 0.06 | 0.32 ± 0.09 | 0.26 ± 0.06 | 0.29 ± 0.07 | 0.35 ± 0.12 | 0.32 ± 0.05 | 0.18 ± 0.05 | 0.26 ± 0.10 | 0.31 ± 0.11 | 0.15 ± 0.05 | 0.20 ± 0.07 |
| WRIST | FGA 1 | 0.13 ± 0.07 | 0.15 ± 0.05 | 0.15 ± 0.08 | 0.14 ± 0.04 | 0.18 ± 0.05 | 0.16 ± 0.07 | 0.12 ± 0.04 | 0.19 ± 0.07 | 0.14 ± 0.06 | 0.14 ± 0.05 | 0.17 ± 0.05 | 0.14 ± 0.04 |
| | FGA 2 | 0.27 ± 0.07 | 0.32 ± 0.09 | 0.36 ± 0.13 | 0.34 ± 0.06$^{**}$ | 0.33 ± 0.10 | 0.42 ± 0.11 | 0.35 ± 0.12 | 0.45 ± 0.12$^{*}$ | 0.34 ± 0.07 | 0.36 ± 0.12 | 0.37 ± 0.08 | 0.36 ± 0.09 |
| | FGA 3 | 0.23 ± 0.15 | 0.26 ± 0.10$^{*}$ | 0.25 ± 0.16 | 0.18 ± 0.20 | 0.18 ± 0.07 | 0.18 ± 0.14 | 0.20 ± 0.18 | 0.31 ± 0.15$^{**}$ | 0.26 ± 0.22 | 0.16 ± 0.13 | 0.19 ± 0.06 | 0.17 ± 0.09 |
| | FGA 4 | 0.19 ± 0.10$^{*}$ | 0.22 ± 0.08$^{***}$ | 0.22 ± 0.12$^{**}$ | 0.13 ± 0.05 | 0.15 ± 0.03 | 0.13 ± 0.04 | 0.17 ± 0.09$^{*}$ | 0.30 ± 0.14$^{***}$ | 0.22 ± 0.11$^{**}$ | 0.11 ± 0.02 | 0.17 ± 0.04 | 0.15 ± 0.04 |
| | FGA 5 | 0.16 ± 0.06 | 0.21 ± 0.08 | 0.19 ± 0.07 | 0.18 ± 0.06 | 0.24 ± 0.11 | 0.21 ± 0.06 | 0.16 ± 0.07 | 0.24 ± 0.09 | 0.20 ± 0.08 | 0.18 ± 0.06 | 0.27 ± 0.11 | 0.23 ± 0.10 |
| | FGA 6 | 0.26 ± 0.11 | 0.36 ± 0.12$^{***}$ | 0.33 ± 0.13 | 0.25 ± 0.12 | 0.25 ± 0.02 | 0.27 ± 0.09 | 0.22 ± 0.09 | 0.42 ± 0.14 | 0.33 ± 0.09 | 0.22 ± 0.08 | 0.38 ± 0.08 | 0.29 ± 0.08 |
| | FGA 7 | 0.35 ± 0.10 | 0.34 ± 0.11 | 0.36 ± 0.09 | 0.30 ± 0.07 | 0.28 ± 0.09 | 0.30 ± 0.13 | 0.42 ± 0.11 | 0.40 ± 0.12$^{*}$ | 0.37 ± 0.15 | 0.38 ± 0.18 | 0.32 ± 0.08 | 0.32 ± 0.19 |
| | FGA 8 | 0.27 ± 0.17 | 0.28 ± 0.14$^{***}$ | 0.26 ± 0.11 | 0.20 ± 0.07 | 0.16 ± 0.06 | 0.23 ± 0.11 | 0.36 ± 0.40 | 0.32 ± 0.12 | 0.32 ± 0.28 | 0.25 ± 0.35 | 0.26 ± 0.18 | 0.20 ± 0.18 |
| | FGA 9 | 0.21 ± 0.10 | 0.26 ± 0.10$^{**}$ | 0.25 ± 0.07$^{*}$ | 0.18 ± 0.06 | 0.18 ± 0.03 | 0.19 ± 0.06 | 0.25 ± 0.10 | 0.26 ± 0.09 | 0.21 ± 0.14 | 0.21 ± 0.12 | 0.30 ± 0.28 | 0.16 ± 0.07 |
| | FGA 10 | 0.47 ± 0.23$^{*}$ | 0.52 ± 0.15 | 0.44 ± 0.16 | 0.33 ± 0.12 | 0.57 ± 0.22 | 0.40 ± 0.13 | 0.43 ± 0.34 | 0.58 ± 0.32 | 0.49 ± 0.33 | 0.30 ± 0.10 | 0.44 ± 0.15 | 0.36 ± 0.15 |

**SD:** standard deviation. **IMU:** inertial measurement unit. **FGA:** Functional Gait Assessment scale. **Vert:** vertical acceleration. **AP:** antero-posterior acceleration. **Lat:** lateral acceleration. **FGA1:** Gait level surface. **FGA2:** Change in gait speed. **FGA3:** Gait with horizontal head turns. **FGA4:** Gait with vertical head turns. **FGA5:** Gait and pivot turn. **FGA6:** Step over obstacle. **FGA7:** Gait with narrow base of support. **FGA8:** Gait with eyes closed. **FGA9:** Ambulating backwards. **FGA10:** Steps. Blue asterisks indicate significantly greater values for the healthy control group. Red asterisks indicate significantly greater values for the SCDS group. * $p<0.05$, ** $p<0.01$, *** $p<0.001$.

**Supplementary Table 7:** Mean, standard deviation, and coefficient of variation of gait cycle (stride) duration, estimated step time and length asymmetry and task-specific gait speeds during different items of the Functional Gait Assessment scale.

| FGA tasks | SCDS Group | | | | | | Healthy Control Group | | | | | |
|---|---|---|---|---|---|---|---|---|---|---|---|---|
| | **Gait Cycle Duration** (sec) | | | **Step asymmetry** (ratio) | | **Task-specific gait speed** (m/s)† | **Gait Cycle Duration** (sec) | | | **Step asymmetry** (ratio) | | **Task-specific gait speed** (m/s)† |
| | **Mean** | **SD** | **CV** | **Length** | **Time** | **Mean** | **Mean** | **SD** | **CV** | **Length** | **Time** | **Mean** |
| **FGA 1** | 1.14 ± 0.08* | 0.05 ± 0.03 | 0.04 ± 0.02 | 0.03 ± 0.07 | -0.01 ± 0.07 | 1.11 ± 0.20 | 1.09 ± 0.08 | 0.07 ± 0.05 | 0.06 ± 0.04 | 0.01 ± 0.06 | 0.02 ± 0.06 | 1.27 ± 0.22* |
| **FGA 2** | 1.24 ± 0.14 | 0.34 ± 0.09 | 0.27 ± 0.06 | -0.03 ± 0.08 | -0.03 ± 0.32 | 0.55 ± 0.14 | 1.17 ± 0.10 | 0.27 ± 0.12 | 0.23 ± 0.08 | -0.01 ± 0.10 | -1.40 ± 5.76 | 0.61 ± 0.13 |
| **FGA 3** | 1.33 ± 0.24* | 0.09 ± 0.12* | 0.06 ± 0.05* | -0.04 ± 0.10 | 0.00 ± 0.09 | 0.46 ± 0.11 | 1.17 ± 0.14 | 0.04 ± 0.01 | 0.03 ± 0.01 | 0.01 ± 0.05 | -0.01 ± 0.08 | 0.54 ± 0.15 |
| **FGA 4** | 1.28 ± 0.16** | 0.05 ± 0.04 | 0.04 ± 0.02 | -0.06 ± 0.23 | -0.83 ± 3.17 | 0.45 ± 0.10 | 1.14 ± 0.12 | 0.04 ± 0.01 | 0.03 ± 0.01 | -0.05 ± 0.12 | 0.02 ± 0.05 | 0.57 ± 0.15* |
| **FGA 5** | 1.24 ± 0.15 | 0.13 ± 0.14 | 0.10 ± 0.09 | 0.04 ± 0.07 | 0.00 ± 0.07 | 0.48 ± 0.09 | 1.15 ± 0.10 | 0.10 ± 0.12 | 0.08 ± 0.09 | 0.04 ± 0.08 | 0.03 ± 0.29 | 0.52 ± 0.13 |
| **FGA 6** | 1.35 ± 0.15** | 0.27 ± 0.12*** | 0.20 ± 0.08** | -0.02 ± 0.15 | -3.21 ± 12.39 | 0.55 ± 0.13 | 1.20 ± 0.12 | 0.14 ± 0.07 | 0.12 ± 0.05 | 0.01 ± 0.09 | -0.06 ± 0.11 | 0.64 ± 0.14 |
| **FGA 7** | 2.61 ± 0.61* | 0.39 ± 0.31 | 0.14 ± 0.10 | -0.02 ± 0.12 | -2.68 ± 7.99 | 0.28 ± 0.06 | 2.17 ± 0.43 | 0.22 ± 0.22 | 0.10 ± 0.09 | 0.05 ± 0.10 | -6.42 ± 16.24 | 0.30 ± 0.09 |
| **FGA 8** | 1.59 ± 0.32** | 0.13 ± 0.14* | 0.07 ± 0.06 | -0.02 ± 0.10 | -3.43 ± 12.36 | 0.55 ± 0.20 | 1.32 ± 0.16 | 0.07 ± 0.02 | 0.06 ± 0.02 | 0.01 ± 0.08 | -0.23 ± 0.38 | 0.76 ± 0.17** |
| **FGA 9** | 1.51 ± 0.24*** | 0.09 ± 0.04 | 0.06 ± 0.02 | 0.01 ± 0.09 | -0.04 ± 0.29 | 0.42 ± 0.17 | 1.23 ± 0.14 | 0.08 ± 0.06 | 0.07 ± 0.05 | 0.01 ± 0.07 | 0.00 ± 0.21 | 0.51 ± 0.08 |
| **FGA 10** | 1.26 ± 0.24*** | 0.13 ± 0.07 | 0.10 ± 0.04 | -0.03 ± 0.07 | 0.00 ± 0.14 | 0.43 ± 0.08 | 1.03 ± 0.12 | 0.12 ± 0.04 | 0.12 ± 0.04 | 0.01 ± 0.09 | 0.00 ± 0.21 | 0.54 ± 0.09** |

Values presented are mean ± standard deviation. SD: standard deviation. CV: Coefficient of variation. FGA: Functional Gait Assessment scale. FGA1: Gait level surface; FGA2: Change in gait speed; FGA3: Gait with horizontal head turns; FGA4: Gait with vertical head turns; FGA5: Gait and pivot turn; FGA6: Step over obstacle; FGA7: Gait with narrow base of support; FGA8: Gait with eyes closed; FGA9: Ambulating backwards; FGA10: Steps. Red asterisks indicate significantly greater values for the SCDS group. Blue asterisks indicate significantly greater values for the healthy control group. * $p<0.05$, ** $p<0.01$, *** $p<0.001$. †Gait speed values for FGA tasks 1 and 8 were calculated by dividing the distance by the completion time, and for the remaining tasks gait speed was estimated using ankle IMU data.

**Supplementary Table 8:** Mean ± SD of 'range' of 3D angular velocities and 3D linear accelerations during 10 gait tasks of the FGA scale, recorded from IMUs on the back and contra-lesional ankle.

| | | Range of Angular Velocities (deg/s) | | | | | | Range of Linear Accelerations (g) | | | | | |
|---|---|---|---|---|---|---|---|---|---|---|---|---|---|
| IMU | FGA Tasks | SCDS Group | | | Control Group | | | SCDS Group | | | Control Group | | |
| | | Pitch | Yaw | Roll | Pitch | Yaw | Roll | Vert | AP | Lat | Vert | AP | Lat |
| BACK | FGA 1 | 80 ± 69 | 73 ± 38 | 38 ± 15 | 70 ± 27 | 65 ± 16 | 40 ± 13 | 0.76 ± 0.16 | 0.42 ± 0.12 | 0.35 ± 0.18 | 0.87 ± 0.20 | 0.43 ± 0.11 | 0.38 ± 0.13 |
| | FGA 2 | 80 ± 61 | 69 ± 27 | 35 ± 12 | 73 ± 25 | 69 ± 18 | 31 ± 11 | 0.80 ± 0.29 | 0.36 ± 0.11 | 0.40 ± 0.14 | 0.92 ± 0.18 | 0.36 ± 0.11 | 0.36 ± 0.16 |
| | FGA 3 | 72 ± 57 | 58 ± 21 | 33 ± 10 | 57 ± 19 | 58 ± 20 | 31 ± 14 | 0.64 ± 0.19 | 0.35 ± 0.09 | 0.31 ± 0.11 | 0.74 ± 0.19 | 0.36 ± 0.08 | 0.28 ± 0.08 |
| | FGA 4 | 75 ± 61 | 58 ± 26 | 32 ± 12 | 57 ± 14 | 59 ± 19 | 31 ± 12 | 0.66 ± 0.21 | 0.37 ± 0.08 | 0.29 ± 0.10 | 0.74 ± 0.15 | 0.35 ± 0.06 | 0.29 ± 0.09 |
| | FGA 5 | 61 ± 40 | 63 ± 25 | 31 ± 12 | 58 ± 14 | 74 ± 21 | 33 ± 11 | 0.61 ± 0.16 | 0.37 ± 0.10 | 0.29 ± 0.09 | 0.71 ± 0.15 | 0.36 ± 0.05 | 0.31 ± 0.10 |
| | FGA 6 | 86 ± 91 | 63 ± 28 | 37 ± 14 | 67 ± 29 | 64 ± 16 | 36 ± 10 | 0.72 ± 0.29 | 0.49 ± 0.14 | 0.36 ± 0.17 | 0.79 ± 0.20 | 0.55 ± 0.19 | 0.39 ± 0.12 |
| | FGA 7 | 32 ± 15* | 34 ± 9 | 26 ± 15 | 24 ± 6 | 34 ± 11 | 20 ± 7 | 0.18 ± 0.07 | 0.18 ± 0.08 | 0.19 ± 0.08 | 0.19 ± 0.13 | 0.16 ± 0.05 | 0.16 ± 0.05 |
| | FGA 8 | 72 ± 147 | 49 ± 16 | 22 ± 10 | 40 ± 16 | 58 ± 15 | 21 ± 8 | 0.28 ± 0.14 | 0.23 ± 0.23 | 0.24 ± 0.06 | 0.42 ± 0.16* | 0.23 ± 0.08 | 0.22 ± 0.06 |
| | FGA 9 | 68 ± 70 | 52 ± 23 | 26 ± 11 | 69 ± 29 | 62 ± 19 | 22 ± 7 | 0.28 ± 0.17 | 0.19 ± 0.13 | 0.29 ± 0.11 | 0.45 ± 0.18* | 0.20 ± 0.08 | 0.33 ± 0.10 |
| | FGA 10 | 64 ± 51 | 45 ± 23 | 32 ± 13 | 64 ± 36 | 55 ± 22 | 30 ± 16 | 0.66 ± 0.13 | 0.30 ± 0.10 | 0.29 ± 0.09 | 0.87 ± 0.31* | 0.35 ± 0.12 | 0.38 ± 0.10* |
| CONTRA-LESIONAL ANKLE | FGA 1 | 583 ± 69 | 221 ± 88 | 125 ± 58 | 617 ± 87 | 234 ± 85 | 146 ± 77 | 2.12 ± 0.41 | 3.87 ± 1.60 | 1.16 ± 0.42 | 2.17 ± 0.43 | 4.03 ± 1.04 | 1.18 ± 0.38 |
| | FGA 2 | 500 ± 72 | 164 ± 59 | 94 ± 41 | 517 ± 77 | 149 ± 32 | 121 ± 59 | 1.66 ± 0.37 | 3.08 ± 0.97 | 0.93 ± 0.44 | 1.70 ± 0.39 | 2.97 ± 0.61 | 0.85 ± 0.21 |
| | FGA 3 | 501 ± 95 | 197 ± 79 | 102 ± 51 | 580 ± 103* | 211 ± 70 | 137 ± 75 | 1.69 ± 0.48 | 3.03 ± 1.23 | 0.91 ± 0.42 | 2.00 ± 0.52 | 4.04 ± 1.36* | 1.03 ± 0.40 |
| | FGA 4 | 517 ± 80 | 195 ± 77 | 101 ± 47 | 594 ± 90* | 218 ± 64 | 138 ± 73 | 1.75 ± 0.52 | 3.22 ± 1.48 | 0.86 ± 0.36 | 2.13 ± 0.57 | 4.20 ± 1.16* | 1.22 ± 0.30** |
| | FGA 5 | 509 ± 87 | 199 ± 60 | 106 ± 48 | 543 ± 74 | 186 ± 60 | 122 ± 60 | 1.76 ± 0.39 | 3.21 ± 0.87 | 1.01 ± 0.33 | 1.88 ± 0.41 | 3.31 ± 1.14 | 1.01 ± 0.34 |
| | FGA 6 | 460 ± 88 | 187 ± 77 | 90 ± 39 | 556 ± 96** | 187 ± 55 | 128 ± 71 | 1.63 ± 0.65 | 3.09 ± 1.46 | 0.93 ± 0.66 | 1.96 ± 0.57 | 3.02 ± 1.12 | 1.07 ± 0.43 |
| | FGA 7 | 283 ± 81 | 135 ± 46 | 73 ± 25 | 330 ± 83 | 160 ± 59 | 99 ± 30* | 0.86 ± 0.42 | 2.05 ± 0.91 | 0.60 ± 0.16 | 1.02 ± 0.42 | 2.03 ± 0.85 | 0.74 ± 0.25 |
| | FGA 8 | 368 ± 104 | 116 ± 62 | 71 ± 32 | 471 ± 67** | 154 ± 67 | 104 ± 53* | 1.13 ± 0.54 | 1.77 ± 1.10 | 0.49 ± 0.24 | 1.55 ± 0.33* | 2.32 ± 0.81 | 0.66 ± 0.30 |
| | FGA 9 | 325 ± 62 | 111 ± 37 | 75 ± 32 | 388 ± 77* | 172 ± 56*** | 95 ± 30 | 0.85 ± 0.30 | 1.67 ± 0.79 | 0.58 ± 0.27 | 1.11 ± 0.37* | 2.45 ± 0.63** | 0.87 ± 0.30** |
| | FGA 10 | 304 ± 52 | 124 ± 38 | 78 ± 34 | 343 ± 66 | 138 ± 54 | 88 ± 45 | 1.24 ± 0.34 | 2.00 ± 0.81 | 0.74 ± 0.34 | 1.51 ± 0.36* | 2.75 ± 0.81* | 0.98 ± 0.27* |

**SD:** standard deviation. **IMU:** inertial measurement unit. **FGA:** Functional Gait Assessment scale. **Vert:** vertical acceleration. **AP:** antero-posterior acceleration. **Lat:** lateral acceleration. **FGA1:** Gait level surface. **FGA2:** Change in gait speed. **FGA3:** Gait with horizontal head turns. **FGA4:** Gait with vertical head turns. **FGA5:** Gait and pivot turn. **FGA6:** Step over obstacle. **FGA7:** Gait with narrow base of support. **FGA8:** Gait with eyes closed. **FGA9:** Ambulating backwards. **FGA10:** Steps. Blue asterisks indicate significantly greater values for the healthy control group. Red asterisks indicate significantly greater values for the SCDS group. * p<0.05, ** p<0.01, *** p<0.001.

**Supplementary Table 9:** Mean ± SD of 'standard deviation' of 3D angular velocities and 3D linear accelerations during 10 gait tasks of the FGA scale, recorded from IMUs on the back and contra-lesional ankle.

| | | Standard Deviation of Angular Velocities (deg/s) | | | | | | Standard Deviation of Linear Accelerations (g) | | | | | |
|---|---|---|---|---|---|---|---|---|---|---|---|---|---|
| **IMU** | **FGA Tasks** | **SCDS Group** | | | **Control Group** | | | **SCDS Group** | | | **Control Group** | | |
| | | **Pitch** | **Yaw** | **Roll** | **Pitch** | **Yaw** | **Roll** | **Vert** | **AP** | **Lat** | **Vert** | **AP** | **Lat** |
| **BACK** | **FGA 1** | 10 ± 3 | 9 ± 2 | 5 ± 1 | 12 ± 4 | 10 ± 2 | 5 ± 1 | 0.07 ± 0.02 | 0.05 ± 0.01 | 0.05 ± 0.01 | 0.09 ± 0.02* | 0.05 ± 0.02 | 0.05 ± 0.01 |
| | **FGA 2** | 24 ± 17 | 17 ± 6 | 10 ± 3* | 21 ± 6 | 17 ± 5 | 8 ± 2 | 0.18 ± 0.05 | 0.10 ± 0.03 | 0.11 ± 0.04 | 0.19 ± 0.04 | 0.10 ± 0.03 | 0.10 ± 0.03 |
| | **FGA 3** | 13 ± 6 | 15 ± 5* | 8 ± 2*** | 11 ± 2 | 12 ± 3 | 6 ± 1 | 0.08 ± 0.02 | 0.06 ± 0.02* | 0.07 ± 0.02* | 0.07 ± 0.02 | 0.05 ± 0.01 | 0.06 ± 0.01 |
| | **FGA 4** | 15 ± 5* | 10 ± 2* | 6 ± 2** | 12 ± 3 | 8 ± 2 | 5 ± 1 | 0.08 ± 0.02 | 0.09 ± 0.03** | 0.06 ± 0.02* | 0.07 ± 0.02 | 0.06 ± 0.02 | 0.05 ± 0.01 |
| | **FGA 5** | 11 ± 4 | 13 ± 5 | 6 ± 2 | 12 ± 3 | 19 ± 10* | 6 ± 2 | 0.08 ± 0.03 | 0.06 ± 0.02 | 0.05 ± 0.02 | 0.09 ± 0.03 | 0.06 ± 0.02 | 0.06 ± 0.02 |
| | **FGA 6** | 21 ± 13 | 15 ± 3 | 9 ± 3 | 18 ± 6 | 18 ± 5 | 8 ± 2 | 0.12 ± 0.03 | 0.11 ± 0.03 | 0.08 ± 0.02 | 0.14 ± 0.04 | 0.11 ± 0.03 | 0.08 ± 0.02 |
| | **FGA 7** | 9 ± 5* | 10 ± 4 | 11 ± 7* | 7 ± 2 | 8 ± 3 | 7 ± 4 | 0.06 ± 0.02 | 0.06 ± 0.03 | 0.08 ± 0.04 | 0.05 ± 0.02 | 0.05 ± 0.03 | 0.06 ± 0.03 |
| | **FGA 8** | 12 ± 15 | 9 ± 2 | 5 ± 3 | 8 ± 3 | 7 ± 2 | 4 ± 1 | 0.05 ± 0.01 | 0.05 ± 0.02* | 0.05 ± 0.02 | 0.06 ± 0.02 | 0.04 ± 0.01 | 0.04 ± 0.01 |
| | **FGA 9** | 12 ± 12 | 8 ± 1 | 5 ± 1 | 10 ± 4 | 9 ± 3 | 5 ± 1 | 0.05 ± 0.02 | 0.05 ± 0.02 | 0.05 ± 0.01 | 0.06 ± 0.03 | 0.05 ± 0.02 | 0.05 ± 0.01 |
| | **FGA 10** | 25 ± 22 | 24 ± 5 | 9 ± 2 | 19 ± 6 | 31 ± 7** | 11 ± 4 | 0.17 ± 0.06 | 0.12 ± 0.05 | 0.09 ± 0.02 | 0.21 ± 0.05 | 0.12 ± 0.03 | 0.10 ± 0.02 |
| **CONTRA-LESIONAL ANKLE** | **FGA 1** | 29±12 | 25±7 | 11±2 | 40±11* | 28±6 | 15±5** | 0.14 ± 0.05 | 0.27 ± 0.09 | 0.13 ± 0.02 | 0.21 ± 0.04*** | 0.35 ± 0.09* | 0.15 ± 0.04 |
| | **FGA 2** | 88±16 | 40±11 | 19±5 | 87±19 | 38±8 | 23±9 | 0.37 ± 0.08 | 0.60 ± 0.17 | 0.21 ± 0.07 | 0.39 ± 0.08 | 0.60 ± 0.12 | 0.22 ± 0.06 |
| | **FGA 3** | 32±13 | 31±6 | 14±3 | 27±7 | 28±6 | 13±3 | 0.14 ± 0.03 | 0.29 ± 0.06 | 0.14 ± 0.03 | 0.15 ± 0.04 | 0.27 ± 0.08 | 0.14 ± 0.03 |
| | **FGA 4** | 29±11 | 27±4 | 12±2 | 27±6 | 26±5 | 12±3 | 0.14 ± 0.03 | 0.27 ± 0.04 | 0.13 ± 0.02 | 0.15 ± 0.04 | 0.26 ± 0.08 | 0.13 ± 0.03 |
| | **FGA 5** | 44±24 | 29±8 | 13±5 | 50±22 | 30±5 | 16±8 | 0.18 ± 0.07 | 0.29 ± 0.08 | 0.13 ± 0.04 | 0.22 ± 0.06 | 0.34 ± 0.10 | 0.15 ± 0.04 |
| | **FGA 6** | 75±29 | 35±6 | 19±4 | 79±29 | 38±10 | 24±8* | 0.30 ± 0.07 | 0.45 ± 0.09 | 0.17 ± 0.04 | 0.35 ± 0.10 | 0.54 ± 0.16 | 0.20 ± 0.04* |
| | **FGA 7** | 34±10 | 31±6 | 12±2 | 32±10 | 30±7 | 13±5 | 0.14 ± 0.03 | 0.24 ± 0.07 | 0.13 ± 0.03 | 0.14 ± 0.05 | 0.25 ± 0.08 | 0.13 ± 0.03 |
| | **FGA 8** | 33±11 | 24±7 | 10±2 | 32±15 | 24±7 | 12±5 | 0.13 ± 0.02 | 0.21 ± 0.05 | 0.10 ± 0.02 | 0.15 ± 0.07 | 0.23 ± 0.09 | 0.11 ± 0.03 |
| | **FGA 9** | 23±3 | 22±5 | 9±2 | 28±13 | 27±8 | 12±4* | 0.10 ± 0.02 | 0.20 ± 0.03 | 0.10 ± 0.03 | 0.13 ± 0.06 | 0.23 ± 0.08 | 0.13 ± 0.04* |
| | **FGA 10** | 70±9 | 37±8 | 19±4 | 85±18** | 42±9 | 27±8** | 0.40 ± 0.09 | 0.46 ± 0.11 | 0.20 ± 0.05 | 0.52 ± 0.11** | 0.57 ± 0.11* | 0.25 ± 0.05** |

**SD:** standard deviation. **IMU:** inertial measurement unit. **FGA:** Functional Gait Assessment scale. **Vert:** vertical acceleration. **AP:** antero-posterior acceleration. **Lat:** lateral acceleration. **FGA1:** Gait level surface. **FGA2:** Change in gait speed. **FGA3:** Gait with horizontal head turns. **FGA4:** Gait with vertical head turns. **FGA5:** Gait and pivot turn. **FGA6:** Step over obstacle. **FGA7:** Gait with narrow base of support. **FGA8:** Gait with eyes closed. **FGA9:** Ambulating backwards. **FGA10:** Steps. Blue asterisks indicate significantly greater values for the healthy control group. Red asterisks indicate significantly greater values for the SCDS group. * p<0.05, ** p<0.01, *** p<0.001.

**Supplementary Table 10:** Mean ± SD of ‘variability’ of 3D angular velocities and 3D linear accelerations during 10 gait tasks of the FGA scale, recorded from IMUs on the back and contra-lesional ankle.

| | | Variability of Angular Velocities | | | | | | Variability of Linear Accelerations | | | | | |
|---|---|---|---|---|---|---|---|---|---|---|---|---|---|
| IMU | FGA Tasks | SCDS Group | | | Control Group | | | SCDS Group | | | Control Group | | |
| | | Pitch | Yaw | Roll | Pitch | Yaw | Roll | Vert | AP | Lat | Vert | AP | Lat |
| BACK | FGA 1 | 0.16 ± 0.06 | 0.15 ± 0.07 | 0.15 ± 0.07 | 0.18 ± 0.05 | 0.17 ± 0.05 | 0.14 ± 0.05 | 0.10 ± 0.03 | 0.12 ± 0.03 | 0.16 ± 0.08 | 0.10 ± 0.02 | 0.13 ± 0.03 | 0.14 ± 0.04 |
| | FGA 2 | 0.32 ± 0.09 | 0.26 ± 0.06 | 0.31 ± 0.11 | 0.30 ± 0.10 | 0.26 ± 0.08 | 0.27 ± 0.08 | 0.24 ± 0.05 | 0.29 ± 0.07 | 0.27 ± 0.05 | 0.21 ± 0.04 | 0.27 ± 0.04 | 0.28 ± 0.07 |
| | FGA 3 | 0.21 ± 0.08 | 0.29 ± 0.12 | 0.27 ± 0.12 | 0.20 ± 0.05 | 0.23 ± 0.10 | 0.22 ± 0.09 | 0.13 ± 0.05* | 0.18 ± 0.07* | 0.24 ± 0.09 | 0.10 ± 0.02 | 0.14 ± 0.03 | 0.21 ± 0.06 |
| | FGA 4 | 0.25 ± 0.08 | 0.19 ± 0.07 | 0.25 ± 0.17 | 0.21 ± 0.06 | 0.15 ± 0.05 | 0.18 ± 0.07 | 0.13 ± 0.04** | 0.26 ± 0.10** | 0.21 ± 0.07* | 0.09 ± 0.01 | 0.18 ± 0.06 | 0.17 ± 0.04 |
| | FGA 5 | 0.21 ± 0.08 | 0.22 ± 0.09 | 0.20 ± 0.09 | 0.21 ± 0.08 | 0.26 ± 0.11 | 0.18 ± 0.04 | 0.14 ± 0.06 | 0.16 ± 0.06 | 0.19 ± 0.07 | 0.13 ± 0.03 | 0.17 ± 0.03 | 0.18 ± 0.04 |
| | FGA 6 | 0.31 ± 0.13 | 0.27 ± 0.08 | 0.28 ± 0.11 | 0.28 ± 0.07 | 0.28 ± 0.06 | 0.25 ± 0.08 | 0.17 ± 0.05 | 0.23 ± 0.07 | 0.24 ± 0.06 | 0.18 ± 0.04 | 0.20 ± 0.03 | 0.21 ± 0.04 |
| | FGA 7 | 0.30 ± 0.09 | 0.30 ± 0.09 | 0.43 ± 0.12 | 0.27 ± 0.05 | 0.25 ± 0.07 | 0.36 ± 0.13 | 0.32 ± 0.07 | 0.34 ± 0.17 | 0.42 ± 0.09 | 0.30 ± 0.07 | 0.33 ± 0.18 | 0.38 ± 0.17 |
| | FGA 8 | 0.26 ± 0.11 | 0.20 ± 0.10* | 0.25 ± 0.11 | 0.21 ± 0.08 | 0.14 ± 0.05 | 0.22 ± 0.08 | 0.21 ± 0.07** | 0.29 ± 0.18** | 0.21 ± 0.08 | 0.14 ± 0.04 | 0.17 ± 0.05 | 0.18 ± 0.05 |
| | FGA 9 | 0.19 ± 0.05 | 0.19 ± 0.08 | 0.25 ± 0.13 | 0.15 ± 0.05 | 0.15 ± 0.05 | 0.23 ± 0.08 | 0.21 ± 0.07** | 0.28 ± 0.09 | 0.17 ± 0.06 | 0.15 ± 0.04 | 0.28 ± 0.08 | 0.16 ± 0.05 |
| | FGA 10 | 0.40 ± 0.10 | 0.62 ± 0.32 | 0.32 ± 0.12 | 0.33 ± 0.10 | 0.63 ± 0.23 | 0.46 ± 0.33 | 0.26 ± 0.07 | 0.43 ± 0.17 | 0.33 ± 0.06* | 0.27 ± 0.09 | 0.37 ± 0.11 | 0.28 ± 0.05 |
| CONTRA-LESIONAL ANKLE | FGA 1 | 0.05 ± 0.02 | 0.13 ± 0.05 | 0.11 ± 0.05 | 0.07 ± 0.03 | 0.13 ± 0.03 | 0.11 ± 0.04 | 0.07 ± 0.03 | 0.08 ± 0.04 | 0.12 ± 0.04 | 0.10 ± 0.02** | 0.09 ± 0.02 | 0.13 ± 0.04 |
| | FGA 2 | 0.18 ± 0.04 | 0.26 ± 0.07 | 0.22 ± 0.07 | 0.17 ± 0.04 | 0.26 ± 0.07 | 0.21 ± 0.06 | 0.23 ± 0.04 | 0.20 ± 0.05 | 0.25 ± 0.09 | 0.24 ± 0.06 | 0.21 ± 0.05 | 0.27 ± 0.07 |
| | FGA 3 | 0.07 ± 0.05 | 0.18 ± 0.08 | 0.16 ± 0.08* | 0.05 ± 0.01 | 0.14 ± 0.03 | 0.11 ± 0.04 | 0.10 ± 0.06 | 0.12 ± 0.07** | 0.18 ± 0.08 | 0.08 ± 0.02 | 0.07 ± 0.02 | 0.14 ± 0.03 |
| | FGA 4 | 0.06 ± 0.03 | 0.16 ± 0.06* | 0.13 ± 0.05* | 0.05 ± 0.01 | 0.12 ± 0.02 | 0.10 ± 0.04 | 0.09 ± 0.06 | 0.11 ± 0.06** | 0.18 ± 0.10*** | 0.07 ± 0.01 | 0.06 ± 0.01 | 0.11 ± 0.03 |
| | FGA 5 | 0.10 ± 0.07 | 0.15 ± 0.05 | 0.13 ± 0.05 | 0.10 ± 0.05 | 0.18 ± 0.05 | 0.14 ± 0.06 | 0.11 ± 0.05 | 0.10 ± 0.04 | 0.14 ± 0.06 | 0.12 ± 0.04 | 0.11 ± 0.03 | 0.16 ± 0.06 |
| | FGA 6 | 0.18 ± 0.09 | 0.21 ± 0.07 | 0.23 ± 0.07 | 0.14 ± 0.05 | 0.21 ± 0.04 | 0.21 ± 0.08 | 0.22 ± 0.10 | 0.17 ± 0.06 | 0.22 ± 0.07 | 0.19 ± 0.07 | 0.20 ± 0.09 | 0.22 ± 0.09 |
| | FGA 7 | 0.14 ± 0.09 | 0.25 ± 0.08 | 0.18 ± 0.07* | 0.11 ± 0.06 | 0.21 ± 0.06 | 0.13 ± 0.05 | 0.20 ± 0.12 | 0.14 ± 0.06 | 0.22 ± 0.07 | 0.15 ± 0.06 | 0.13 ± 0.03 | 0.19 ± 0.06 |
| | FGA 8 | 0.10 ± 0.07* | 0.24 ± 0.07** | 0.16 ± 0.06 | 0.07 ± 0.03 | 0.17 ± 0.05 | 0.14 ± 0.06 | 0.15 ± 0.09* | 0.15 ± 0.07* | 0.24 ± 0.09* | 0.10 ± 0.03 | 0.11 ± 0.04 | 0.18 ± 0.07 |
| | FGA 9 | 0.07 ± 0.02 | 0.22 ± 0.06** | 0.13 ± 0.04 | 0.07 ± 0.04 | 0.16 ± 0.04 | 0.13 ± 0.03 | 0.13 ± 0.04 | 0.13 ± 0.05* | 0.20 ± 0.06 | 0.12 ± 0.04 | 0.10 ± 0.03 | 0.16 ± 0.05 |
| | FGA 10 | 0.24 ± 0.05 | 0.32 ± 0.10 | 0.27 ± 0.08 | 0.26 ± 0.08 | 0.34 ± 0.10 | 0.35 ± 0.14* | 0.33 ± 0.06 | 0.25 ± 0.07 | 0.30 ± 0.12 | 0.36 ± 0.11 | 0.22 ± 0.05 | 0.26 ± 0.07 |

**SD:** standard deviation. **IMU:** inertial measurement unit. **FGA:** Functional Gait Assessment scale. **Vert:** vertical acceleration. **AP:** antero-posterior acceleration. **Lat:** lateral acceleration. **FGA1:** Gait level surface. **FGA2:** Change in gait speed. **FGA3:** Gait with horizontal head turns. **FGA4:** Gait with vertical head turns. **FGA5:** Gait and pivot turn. **FGA6:** Step over obstacle. **FGA7:** Gait with narrow base of support. **FGA8:** Gait with eyes closed. **FGA9:** Ambulating backwards. **FGA10:** Steps. Blue asterisks indicate significantly greater values for the healthy control group. Red asterisks indicate significantly greater values for the SCDS group. * p<0.05, ** p<0.01, *** p<0.001.

**Supplementary Table 11:** Selection of the top three tasks for each IMU based on the magnitude of dissimilarity between the probability distributions of "Single IMU-Single task" global kinematic scores of healthy and SCDS groups. Values reported for the kinematic scores are mean [±1SEM]. This table corresponds to Steps 1 and 2 of Supplementary Figure 1. According to the EMD index, FGA tasks 9, 8, and 10 were the most frequently appeared tasks among at least 4 out of 6 total IMUs (highlighted in green).

| IMUs | Tasks | Kinematic measures used | Controls Kinematic score | SCDS Kinematic score | p-value | EMD |
|---|---|---|---|---|---|---|
| HEAD | FGA1 | 3D Linear & 3D Rotational Ranges | 60.69 [55.18, 66.21] | 41.68 [36.44, 46.92] | 0.0189 | 15.880 |
| | FGA2 | 3D Linear & 3D Rotational Ranges | 57.62 [54.49, 60.74] | 43.63 [39.38, 47.88] | 0.0134 | 12.447 |
| | FGA3 | 3D Linear & 3D Rotational Ranges | 60.01 [57.41, 62.61] | 40.85 [38.29, 43.40] | 0.0000 | 21.040 |
| | FGA4 | 3D Linear & 3D Rotational Ranges | 54.60 [50.76, 58.44] | 49.09 [46.25, 51.93] | 0.2548 | 5.341 |
| | FGA5 | 3D Linear & 3D Rotational Ranges | 61.43 [57.83, 65.03] | 41.38 [35.83, 46.93] | 0.0056 | 18.822 |
| | FGA6 | 3D Linear & 3D Rotational Ranges | 60.40 [54.92, 66.21] | 40.75 [33.89, 47.61] | 0.0380 | 11.681 |
| | FGA7 | 3D Linear & 3D Rotational Ranges | 53.77 [51.37, 56.17] | 45.90 [43.74, 48.05] | 0.0218 | 9.928 |
| | FGA8 | 3D Linear & 3D Rotational Ranges | 61.41 [58.47, 64.34] | 38.80 [34.98, 42.62] | 0.0001 | 21.529 |
| | FGA9 | 3D Linear & 3D Rotational Ranges | 62.37 [56.21, 68.54] | 38.15 [31.42, 44.88] | 0.0138 | 26.003 |
| | FGA10 | 3D Linear & 3D Rotational Ranges | 62.03 [55.78, 68.29] | 37.46 [32.11, 42.80] | 0.0063 | 20.345 |
| BACK | FGA1 | 3D Linear & 3D Rotational Ranges | 53.58 [50.06, 57.10] | 46.82 [43.26, 50.38] | 0.1871 | 6.349 |
| | FGA2 | 3D Linear & 3D Rotational Ranges | 54.06 [50.06, 58.06] | 46.68 [43.66, 49.70] | 0.1512 | 6.692 |
| | FGA3 | 3D Linear & 3D Rotational Ranges | 55.64 [53.54, 57.75] | 47.51 [45.62, 49.40] | 0.0070 | 9.048 |
| | FGA4 | 3D Linear & 3D Rotational Ranges | 53.90 [51.20, 56.60] | 45.95 [42.65, 49.22] | 0.0727 | 10.664 |
| | FGA5 | 3D Linear & 3D Rotational Ranges | 54.39 [49.57, 59.22] | 46.51 [41.90, 51.12] | 0.2437 | 7.448 |
| | FGA6 | 3D Linear & 3D Rotational Ranges | 51.78 [48.78, 54.78] | 47.14 [44.86, 49.42] | 0.2274 | 6.709 |
| | FGA7 | 3D Linear & 3D Rotational Ranges | 57.90 [53.29, 62.50] | 47.93 [43.50, 52.35] | 0.1286 | 9.877 |
| | FGA8 | 3D Linear & 3D Rotational Ranges | 59.63 [55.10, 64.17] | 41.75 [36.96, 46.53] | 0.0124 | 16.945 |
| | FGA9 | 3D Linear & 3D Rotational Ranges | 60.62 [56.16, 65.09] | 39.61 [34.43, 44.79] | 0.0055 | 20.682 |
| | FGA10 | 3D Linear & 3D Rotational Ranges | 58.04 [53.58, 62.46] | 36.88 [32.01, 41.75] | 0.0038 | 20.678 |
| WAIST | FGA1 | 3D Linear & 3D Rotational Ranges | 52.35 [49.16, 55.54] | 45.39 [41.94, 48.84] | 0.1501 | 9.246 |
| | FGA2 | 3D Linear & 3D Rotational Ranges | 53.70 [50.16, 57.23] | 46.32 [43.01, 49.63] | 0.1380 | 5.702 |
| | FGA3 | 3D Linear & 3D Rotational Ranges | 54.85 [50.07, 59.64] | 47.41 [43.00, 51.82] | 0.2606 | 8.675 |
| | FGA4 | 3D Linear & 3D Rotational Ranges | 56.24 [52.14, 60.34] | 46.24 [41.22, 51.26] | 0.1323 | 10.806 |
| | FGA5 | 3D Linear & 3D Rotational Ranges | 54.02 [51.20, 56.83] | 46.03 [43.34, 48.71] | 0.0494 | 7.298 |
| | FGA6 | 3D Linear & 3D Rotational Ranges | 55.13 [50.07, 60.20] | 42.80 [37.38, 48.22] | 0.1068 | 11.144 |
| | FGA7 | 3D Linear & 3D Rotational Ranges | 51.54 [48.65, 54.43] | 45.62 [42.22, 49.03] | 0.1939 | 6.207 |
| | FGA8 | 3D Linear & 3D Rotational Ranges | 63.02 [56.55, 69.49] | 37.86 [31.09, 44.64] | 0.0128 | 15.844 |
| | FGA9 | 3D Linear & 3D Rotational Ranges | 65.60 [59.48, 71.72] | 37.18 [29.70, 44.65] | 0.0074 | 20.991 |
| | FGA10 | 3D Linear & 3D Rotational Ranges | 58.93 [52.47, 65.39] | 36.87 [29.81, 43.92] | 0.0294 | 15.116 |
| WRIST | FGA1 | 3D Linear & 3D Rotational Ranges | 57.68 [55.46, 59.90] | 44.96 [41.71, 48.21] | 0.0038 | 13.694 |
| | FGA2 | 3D Linear & 3D Rotational Ranges | 56.81 [52.51, 61.11] | 45.46 [41.15, 49.78] | 0.0733 | 11.703 |
| | FGA3 | 3D Linear & 3D Rotational Ranges | 55.63 [48.59, 62.67] | 45.33 [38.60, 52.06] | 0.2980 | 17.516 |
| | FGA4 | 3D Linear & 3D Rotational Ranges | 59.03 [54.09, 63.96] | 44.54 [38.44, 50.65] | 0.0752 | 13.357 |
| | FGA5 | 3D Linear & 3D Rotational Ranges | 54.57 [51.39, 57.75] | 44.84 [41.73, 47.94] | 0.0380 | 10.319 |
| | FGA6 | 3D Linear & 3D Rotational Ranges | 56.55 [50.76, 62.35] | 42.09 [35.82, 48.35] | 0.1009 | 13.747 |
| | FGA7 | 3D Linear & 3D Rotational Ranges | 55.59 [49.99, 61.19] | 53.18 [47.82, 58.53] | 0.7563 | 4.480 |
| | FGA8 | 3D Linear & 3D Rotational Ranges | 59.74 [54.36, 65.12] | 37.80 [31.33, 44.28] | 0.0156 | 18.376 |
| | FGA9 | 3D Linear & 3D Rotational Ranges | 61.70 [54.95, 68.45] | 39.22 [33.74, 44.70] | 0.0159 | 24.124 |
| | FGA10 | 3D Linear & 3D Rotational Ranges | 56.54 [50.92, 62.15] | 41.22 [34.10, 48.35] | 0.1017 | 11.967 |
| IPSI-LESIONAL ANKLE | FGA1 | 3D Linear & 3D Rotational Ranges | 59.16 [52.93, 65.40] | 42.38 [35.30, 49.45] | 0.0849 | 10.583 |
| | FGA2 | 3D Linear & 3D Rotational Ranges | 56.68 [50.55, 62.81] | 47.31 [40.67, 53.96] | 0.3059 | 6.033 |
| | FGA3 | 3D Linear & 3D Rotational Ranges | 63.62 [56.70, 70.54] | 40.73 [33.85, 47.61] | 0.0268 | 18.706 |
| | FGA4 | 3D Linear & 3D Rotational Ranges | 62.94 [56.47, 69.41] | 40.63 [33.22, 48.04] | 0.0322 | 21.742 |
| | FGA5 | 3D Linear & 3D Rotational Ranges | 56.82 [51.44, 62.20] | 46.94 [40.56, 53.32] | 0.2455 | 9.244 |
| | FGA6 | 3D Linear & 3D Rotational Ranges | 57.09 [49.86, 64.32] | 41.23 [33.59, 48.86] | 0.1414 | 10.899 |
| | FGA7 | 3D Linear & 3D Rotational Ranges | 56.75 [50.80, 62.71] | 41.44 [33.77, 49.11] | 0.1232 | 13.814 |
| | FGA8 | 3D Linear & 3D Rotational Ranges | 65.62 [60.03, 71.21] | 36.60 [28.19, 45.01] | 0.0087 | 22.899 |
| | FGA9 | 3D Linear & 3D Rotational Ranges | 69.84 [65.16, 74.51] | 33.08 [25.95, 40.21] | 0.0003 | 31.348 |
| | FGA10 | 3D Linear & 3D Rotational Ranges | 64.14 [59.79, 68.49] | 34.04 [27.85, 40.23] | 0.0006 | 25.969 |

**Supplementary Table 11** *(Cont'd):*

| IMUs | Tasks | Kinematic measures used | Controls Kinematic score | SCDS Kinematic score | p-value | EMD |
|---|---|---|---|---|---|---|
| CONTR-LESIONAL ANKLE | FGA1 | 3D Linear & 3D Rotational Ranges | 53.83 [48.42, 59.24] | 45.75 [38.09, 53.41] | 0.3926 | 13.180 |
| | FGA2 | 3D Linear & 3D Rotational Ranges | 53.28 [50.29, 56.28] | 47.34 [44.34, 50.33] | 0.1725 | 6.927 |
| | FGA3 | 3D Linear & 3D Rotational Ranges | 60.76 [54.04, 67.48] | 44.35 [36.75, 51.95] | 0.1155 | 9.357 |
| | FGA4 | 3D Linear & 3D Rotational Ranges | 64.93 [59.14, 70.73] | 40.89 [32.86, 48.92] | 0.0228 | 17.544 |
| | FGA5 | 3D Linear & 3D Rotational Ranges | 52.91 [48.54, 57.28] | 46.82 [43.29, 50.34] | 0.2871 | 6.685 |
| | FGA6 | 3D Linear & 3D Rotational Ranges | 57.86 [53.28, 62.45] | 41.50 [35.66, 47.33] | 0.0364 | 17.977 |
| | FGA7 | 3D Linear & 3D Rotational Ranges | 59.35 [54.73, 63.97] | 43.54 [37.84, 49.24] | 0.0401 | 14.523 |
| | FGA8 | 3D Linear & 3D Rotational Ranges | 65.55 [60.52, 70.57] | 37.30 [29.07, 45.53] | 0.0073 | 19.036 |
| | FGA9 | 3D Linear & 3D Rotational Ranges | 68.10 [62.73, 73.47] | 34.59 [27.82, 41.35] | 0.0009 | 23.749 |
| | FGA10 | 3D Linear & 3D Rotational Ranges | 59.94 [54.71, 65.18] | 40.67 [34.06, 47.29] | 0.0302 | 18.037 |

IMU: inertial measurement unit. SCDS: superior canal dehiscence syndrome. FGA: Functional Gait Assessment scale. FGA1: Gait level surface; FGA2: Change in gait speed; FGA3: Gait with horizontal head turns; FGA4: Gait with vertical head turns; FGA5: Gait and pivot turn; FGA6: Step over obstacle; FGA7: Gait with narrow base of support; FGA8: Gait with eyes closed; FGA9: Ambulating backwards; FGA10: Steps. p-value: significance of between-group differences using an independent permutation test ($\alpha$=0.05). EMD: Earth Mover's Distance index.

**Supplementary Table 12:** The first row of this table corresponds to Step 3.a of Supplementary Figure 1, and shows the total global kinematic score computed from all 6 IMUs, during all 10 FGA tasks. The rest of the rows correspond to Step 3.b of Supplementary Figure 1, and show the optimal global kinematic scores for each IMU using the optimal subset of FGA scale, including FGA tasks 8 and 9. Values reported for the kinematic scores are mean [±1SEM]. According to the EMD index, the optimal global kinematic scores computed for the ankles, waist, and head are the most informative global kinematic scores calculated from individual IMUs using the combination of FGA tasks 8 and 9 (highlighted in green).

| IMUs | FGA Tasks | Kinematic measures used | Controls Kinematic score | SCDS Kinematic score | p-value | EMD |
|---|---|---|---|---|---|---|
| **ALL 6** | ALL | ALL 3D linear & 3D rotational | 61.50 [58.55, 64.45] | 40.07 [35.75, 44.40] | 0.0005 | 21.292 |
| **Ipsi-Ankle** | 8 & 9 | ALL 3D linear & 3D rotational | 63.74 [60.06, 67.43] | 37.82 [31.38, 44.26] | 0.0021 | 23.146 |
| **Contra-Ankle** | 8 & 9 | ALL 3D linear & 3D rotational | 62.04 [58.72, 65.36] | 38.29 [32.21, 44.37] | 0.0023 | 22.586 |
| **Waist** | 8 & 9 | ALL 3D linear & 3D rotational | 62.51 [58.93, 66.10] | 37.66 [32.71, 42.62] | 0.0005 | 25.292 |
| **Head** | 8 & 9 | ALL 3D linear & 3D rotational | 60.26 [56.63, 63.89] | 40.13 [35.49, 44.76] | 0.0026 | 19.393 |
| **Back** | 8 & 9 | ALL 3D linear & 3D rotational | 59.26 [56.01, 62.52] | 42.24 [37.81, 46.67] | 0.0052 | 16.605 |
| **Wrist** | 8 & 9 | ALL 3D linear & 3D rotational | 58.19 [55.16, 61.23] | 41.59 [37.44, 45.74] | 0.0039 | 18.528 |

IMU: inertial measurement unit. SCDS: superior canal dehiscence syndrome. FGA: Functional Gait Assessment scale. FGA8: Gait with eyes closed; FGA9: Ambulating backwards. p-value: significance of between-group differences using an independent permutation test (α=0.05). EMD: Earth Mover's Distance index.

**Supplementary Table 13:** This table corresponds to Step 3.b of Supplementary Figure 1. Calculation of the optimal global kinematic scores for each IMU using a subset of FGA scale, including FGA tasks 8, 9, and 10. Values reported for the kinematic scores are mean [±1SEM]. According to the EMD index, the global kinematic scores computed for the ankles, waist, and head are the most informative global kinematic scores calculated from the combination of FGA tasks 8, 9, and 10 (highlighted in green).

| IMUs | FGA Tasks | Kinematic measures used | Controls Kinematic score | SCDS Kinematic score | p-value | EMD |
|---|---|---|---|---|---|---|
| **Ipsi-Ankle** | 8 & 9 & 10 | ALL 3D linear & 3D rotational | 65.88 [62.51, 69.25] | 34.03 [28.17, 39.90] | 0.0002 | 31.946 |
| **Contra-Ankle** | 8 & 9 & 10 | ALL 3D linear & 3D rotational | 63.69 [60.05, 67.33] | 36.98 [31.28, 42.69] | 0.0008 | 23.534 |
| **Waist** | 8 & 9 & 10 | ALL 3D linear & 3D rotational | 62.51 [58.27, 66.74] | 36.83 [31.80, 41.86] | 0.0008 | 27.212 |
| **Head** | 8 & 9 & 10 | ALL 3D linear & 3D rotational | 61.14 [57.29, 64.99] | 38.92 [34.18, 43.67] | 0.0016 | 20.414 |
| **Back** | 8 & 9 & 10 | ALL 3D linear & 3D rotational | 59.09 [55.52, 62.66] | 40.11 [35.69, 44.52] | 0.0031 | 19.372 |
| **Wrist** | 8 & 9 & 10 | ALL 3D linear & 3D rotational | 60.04 [55.87, 64.20] | 41.09 [35.94, 46.23] | 0.0091 | 19.094 |

IMU: inertial measurement unit. SCDS: superior canal dehiscence syndrome. FGA: Functional Gait Assessment scale. FGA8: Gait with eyes closed; FGA9: Ambulating backwards; FGA10: Steps. p-value: significance of between-group differences using an independent permutation test ($\alpha$=0.05). EMD: Earth Mover's Distance index.

**Supplementary Table 14:** Gait speed scores. Values reported for gait speed scores are mean [±1SEM].

| Measure | Calculated from | Controls Gait speed score | SCDS Gait speed score | p-value | EMD |
|---|---|---|---|---|---|
| **Gait Speed Score** | Normal gait speed over a 10-meter walkway | 70.40 [63.83, 76.97] | 34.66 [26.44, 42.87] | 0.0027 | 16.321 |
| **Gait Speed Score** | Combined FGA tasks 8 & 9 | 72.53 [66.00, 79.05] | 32.96 [23.88, 42.05] | 0.0020 | 19.610 |
| **Gait Speed Score** | Combined FGA tasks 8 & 9 & 10 | 73.90 [67.35, 80.46] | 32.63 [24.17, 41.10] | 0.0009 | 22.723 |

SCDS: superior canal dehiscence syndrome. FGA: Functional Gait Assessment scale. FGA8: Gait with eyes closed; FGA9: Ambulating backwards; FGA10: Steps. p-value: significance of between-group differences using an independent permutation test (α=0.05). EMD: Earth Mover's Distance index.
